# CDetection: CRISPR-Cas12b-based DNA detection with sub-attomolar sensitivity and single-base specificity

**DOI:** 10.1186/s13059-019-1742-z

**Published:** 2019-07-01

**Authors:** Fei Teng, Lu Guo, Tongtong Cui, Xin-Ge Wang, Kai Xu, Qingqin Gao, Qi Zhou, Wei Li

**Affiliations:** 1 State Key Laboratory of Stem Cell and Reproductive Biology, Institute of Zoology, Chinese Academy of Sciences, Beijing, 100101 China; 2 Institute for Stem Cell and Regeneration, Chinese Academy of Sciences, Beijing, 100101 China; 3 University of Chinese Academy of Sciences, Beijing, 100049 China

## Abstract

CRISPR-based nucleic acid detection methods are reported to facilitate rapid and sensitive DNA detection. However, precise DNA detection at the single-base resolution and its wide applications including high-fidelity SNP genotyping remain to be explored. Here we develop a Cas12b-mediated DNA detection (CDetection) strategy, which shows higher sensitivity on examined targets compared with the previously reported Cas12a-based detection platform. Moreover, we show that CDetection can distinguish differences at the single-base level upon combining the optimized tuned guide RNA (tgRNA). Therefore, our findings highlight the high sensitivity and accuracy of CDetection, which provides an efficient and highly practical platform for DNA detection.

**Electronic supplementary material:**

The online version of this article (10.1186/s13059-019-1742-z) contains supplementary material, which is available to authorized users.

## Background

Accurate, rapid, and economical DNA detection method is of great value in clinical diagnostics and quarantine inspection. Recently, CRISPR-Cas-based DNA detection platforms, including SHERLOCK (Cas13) [1–3], DETECTR (Cas12a) [3, 4], and Cas14-DETECTR [5], were developed with high sensitivity and specificity. The SHERLOCK platform utilizes the RNA *trans*-cleavage activity of Cas13 enzymes and combines additional T7 in vitro transcription process, thereby achieving detection of RNA viruses, like Zika virus (ZIKA) and dengue virus (DENV) [2, 3, 6]. Meanwhile, through introducing synthetic mismatches in the guide sequence, this SHERLOCK strategy can discriminate SNPs in human genotyping and cell-free DNA (cfDNA) detection [2, 3, 6]. The Cas12a-DETECTR platform can detect double-stranded DNA (dsDNA) samples directly using its ability to *trans*-cleave single-stranded DNA (ssDNA) reporter [3, 4], yet its accuracy still requires to be improved [4]. More recently, the Cas14-DETECTR platform enables SNP detection in *HERC2* gene [5]. But, Cas14 only binds and cleaves ssDNA, thus requiring an extra step to generate ssDNA from dsDNA to successfully achieve dsDNA detection [5]. Though prior CRISPR-Cas-based nucleic acid detection platforms provide us with sensitive and specific diagnostic methods [2–6], they still require further improvement, and new platforms with prominent features are still desired. Inspired by the ssDNA *trans*-cleavage activity of the Cas12b enzyme [4], we aim to develop a new dsDNA detection strategy combining with our previously reported AaCas12b enzyme [7]. Since the AaCas12b system cleaves dsDNA with high activity and specificity, we speculate that AaCas12b enables direct detection of dsDNA with high sensitivity and accuracy.

## Results and discussion

To test the non-canonical *trans*-cleavage activity of Cas12b (Additional file 1: Figure S1a), we conducted the in vitro ssDNA cleavage assay using Cas12b, Cas12a, and Cas9 with their cognate guide RNAs (gRNAs) (Additional file 2: Table S1). The results showed that AaCas12b [7] and three Cas12a nucleases (ArCas12a, HkCas12a, and PrCas12a) [8] could induce rapid degradation of the single-stranded M13 DNA phage, while SpCas9 could not [4] (Additional file 1: Figure S1b and Additional file 2: Table S1). Meanwhile, AaCas12b and Cas12a proteins degraded M13 phage in the presence of a non-target gRNA (NT-gRNA) and its complementary ssDNA “activator” that shares no sequence homology to the M13 phage genome (Additional file 1: Figure S1c and Additional file 2: Table S1). The non-canonical collateral ssDNA cleavage activity was abolished with catalytically inactive variants, indicating a RuvC domain-dependent cleavage activity (Additional file 1: Figure S2). Collectively, these results reveal that AaCas12b-single guide RNA (sgRNA) complex can acquire RuvC domain-dependent non-specific ssDNA *trans*-cleavage activity once triggered by RNA-guided DNA binding.

Previous study in SHERLOCK [3] indicates that Cas12b might also exhibit biases to different homopolymeric reporters and different buffer composition. So to optimize the Cas12b-mediated DNA detection system, we next profiled the cleavage preference of AaCas12b-sgRNA complex on fluorophore quencher (FQ)-labeled homopolymer reporters. The results showed that AaCas12b preferred thymine polymer (ploy T) as well as poly A and poly C, whereas poly G could hardly be cleaved (Additional file 1: Figure S3a and Additional file 2: Table S1). Meanwhile, the cleavage efficiency could be optimized in NEBuffer™ 2 (Additional file 1: Figure S3b and Additional file 2: Table S2). Afterwards, we performed AaCas12b-mediated cleavage assay using poly T reporter in NEBuffer™ 2. Using sgRNA-complementary on-target ssDNA (OT-ssDNA) and OT-dsDNA, and sgRNA-non-complementary non-target ssDNA (NT-ssDNA) and NT-dsDNA as activators separately, we found OT-ssDNA and OT-dsDNA were able to trigger AaCas12b to cleave the FQ reporter, though OT-dsDNA activator was less efficient than OT-ssDNA (Additional file 1: Figure S3c and Additional file 2: Table S1).

**Fig. 1:**
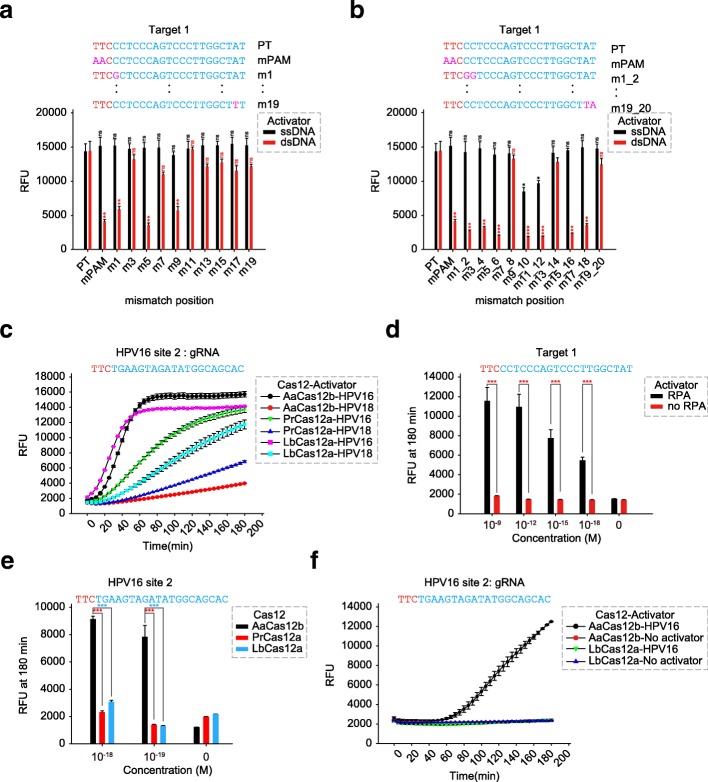
Specificity and sensitivity of Cas12b-mediated DNA detection. **a**, **b** (Upper) Schematics showing mismatched position in the targeting sequence. PAM sequences are colored in red, protospacers are colored in blue, SNPs are colored in pink. (Lower) Characterization of *trans*-cleavage activity of AaCas12b using ssDNA or dsDNA activator with indicated **a** single mismatch or **b** continuous mismatches. Error bars indicate standard errors of the mean (s.e.m.)., *n* = 3. RFU, relative fluorescence units; PT, perfect target; mPAM, mutated PAM. Two-tailed Student’s *t* test is used for significance analysis between each mutated sample and the PT sample. **c** Comparison of the specificity among AaCas12b, PrCas12a, and LbCas12a in dsDNA distinguishability using synthetic HPV16 activator (site 2). Error bars indicate s.e.m., *n* = 3. **d** Comparison of *trans*-cleavage activity and pre-amplification enhanced *trans*-cleavage activity (CDetection) for AaCas12b using dsDNA activator. AaCas12b is incubated with a sgRNA targeting a synthetic dsDNA 1. Error bars indicate s.e.m., *n* = 3. RPA, recombinase polymerase amplification. Two-tailed Student’s *t* test is used for significance analysis between the two samples in each group. **e** Maximum fluorescence signal obtained from AaCas12b-, PrCas12a-, and LbCas12a-based DNA detection with RPA pre-amplification. Cas12 proteins are incubated with their cognate guide RNA (gRNA) targeting a synthetic HPV16 dsDNA (site 2) mixed with background genome. Error bars indicate s.e.m., *n* = 3. Two-tailed Student’s *t* test is used for significance analysis among each other in the group. **f** Fluorescence timecourses obtained from AaCas12b- and LbCas12a-based DNA detection with RPA pre-amplification. Cas12 is incubated with a cognate gRNA targeting a synthetic HPV16 dsDNA (site 2) diluted in human plasma with a final concentration of 10^−18^ M. Error bars indicate s.e.m., *n* = 3. *P < 0.05; **P < 0.01; ***P < 0.001; ns, no significance

We next tested the specificity of *trans*-cleavage activation using either ssDNA or dsDNA activators bearing various mismatches (Additional file 2: Table S1). The results showed that the PAM sequence is critical for the dsDNA activator to trigger AaCas12b *trans*-cleavage activity and is dispensable for the ssDNA activator, and mismatches in dsDNA rather than ssDNA would impede or even abolish the *trans*-cleavage activity of AaCas12b (Fig. 1a, b). Next, we determined the detection sensitivity of AaCas12b-sgRNA-activator system. Without pre-amplification, AaCas12b did not produce a detectable signal when substrate concentrations were lower than 1.6 nM for ssDNA activator and lower than 8 nM for dsDNA activator, respectively (Additional file 1: Figure S4a). Since dsDNA activator possessed a higher specificity (Fig. 1a, b), we engineered the AaCas12b-sgRNA-dsDNA-activator system as a Cas12b-based *D*NA d*etection* (CDetection) platform. To explore the sensitivity of CDetection, we further synthesized one Cauliflower mosaic virus (CaMV) dsDNA and two pairs of human papillomavirus (HPV) dsDNAs (HPV16 and HPV18, site 1 and site 2) [4] as activator for detection reaction (Additional file 2: Table S1). When the input concentration of activator is equal or greater than 10 nM, CDetection could not only produce detectable signals (Additional file 1: Figure S4b-d), but also distinguish two dsDNA viruses, HPV16 and HPV18, in identical group (Additional file 1: Figure S4c, d). Compared with Cas12a-based DNA detection, AaCas12b exhibited higher detection sensitivity than Cas12a did at both detected sites, as CDetection generated a higher signal level and a lower background level (Fig. 1c and Additional file 1: Figure S4e). To further enhance its sensitivity, we performed isothermal amplification by recombinase polymerase amplification (RPA). CDetection combined with RPA enabled substrate detection at 1 attomole (åM) (Fig. 1d and Additional file 1: Figure S4f), which shows higher sensitivity than the Cas12a-based detection platform did [3, 4] (Fig. 1e). Then we chose 12 previously reported targeting sites for comprehensive and unbiased comparison of the sensitivity between AaCas12b- and LbCas12a-based DNA detection (Additional file 2: Table S1). The averaged results (8 of 12 sites) show that CDetection platform possessed a higher sensitivity when compared with LbCas12a-based detection platform except for one site (*DNMT1* site 2) and three sites (*EMX1* site 2, *DNMT1* site 3, and *BRCA1*_3232G) with poor or undetectable signals (Additional file 1: Figure S5).

To explore the applications of CDetection in molecular diagnostics, we tested its feasibility in infectious virus detection. After diluting HPV dsDNAs with human genomic DNAs, we showed that CDetection could identify the diluted substrate at the sub-attomolar magnitude (0.1 åM) (Additional file 1: Figure S6a). The high sensitivity and longevity of AaCas12b in human plasma [7] then urged us to test the application of CDetection in cell-free DNA (cfDNA)-based non-invasive diagnoses. Prior SHERLOCK strategy has achieved cfDNA detection in a simulated condition [2] and using isolated samples [3], but the Cas13-based SHERLOCK requires a T7 in vitro transcription step to achieve DNA detection. To indicate the advantage of CDetection platform in cfDNA detection, we diluted HPV dsDNAs into human plasma and examined the sensitivity of this newly established method. The results showed that CDetection could directly detect the existence of HPV DNAs in human plasma at the concentration of 1 åM (Fig. 1f and Additional file 1: Figure S6b), indicating the possibility to rapidly detect infectious viruses in only one drop of blood. Together, these results demonstrate that CDetection is able to detect target DNA sequences with higher specificity and sensitivity.

**Fig. 2:**
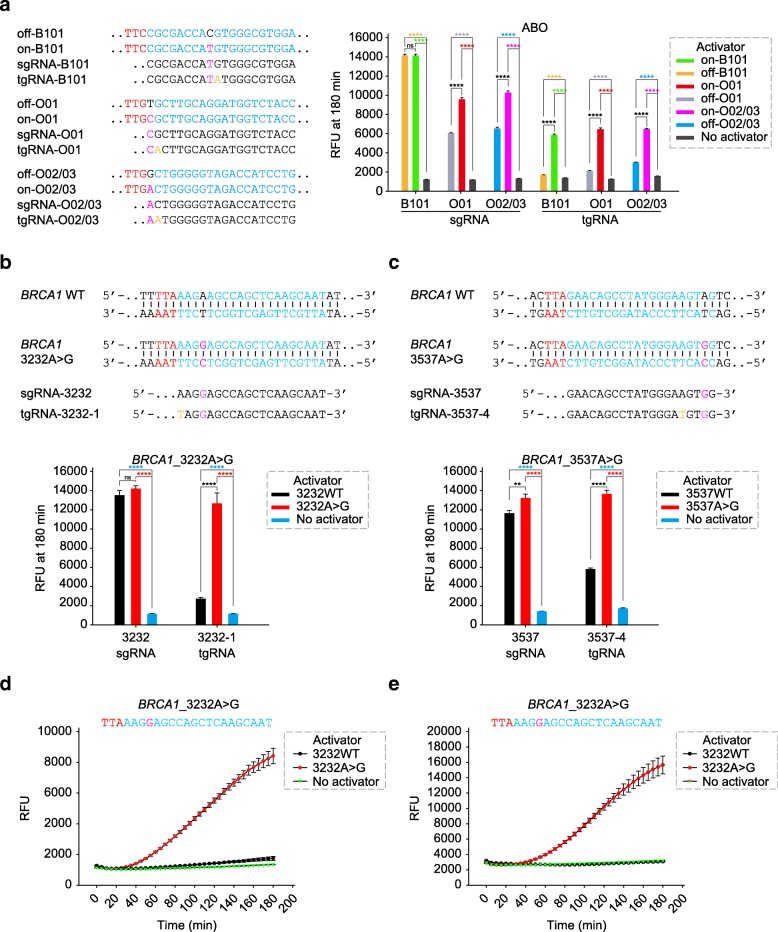
Broad applications for CDetection. **a** (Left) Schematics showing the sequence variation within *ABO* genes and corresponding sgRNAs and tgRNAs. PAM sequences are colored in red, protospacers are colored in blue, SNPs are colored in pink, and base substitution in tgRNAs are colored in orange. (Right) CDetection combined with tgRNA achieve ABO blood genotyping detection with a single-nucleotide-resolution specificity. Error bars indicate standard errors of the mean (s.e.m.), *n* = 3. RFU, relative fluorescence units; tgRNA, tuned guide RNA. Two-tailed Student’s *t* test is used for significance analysis among each other in the group. **b**, **c** (Upper) Schematics showing the sequence variation within *BRCA1* gene and targeting sgRNA and tgRNAs. (Lower) Maximum fluorescence signal showing the specificity of CDetection without RPA for human *BRCA1* **b** 3232A>G and **c** 3537A>G mutation detection using sgRNA and tgRNA. Error bars indicate s.e.m., *n* = 3. Two-tailed Student’s *t* test is used for significance analysis among each other in the group. **d** Fluorescence timecourses showing the sensitivity and specificity of CDetection with RPA for human *BRCA1* 3232A>G mutation detection using tgRNA (3232-1). *BRCA1* wild-type genomic DNAs or 3232A>G mutant genomic DNAs are extracted from cell lines. Error bars indicate s.e.m., *n* = 3. **e** Fluorescence timecourses showing the sensitivity and specificity of CDetection with RPA for human *BRCA1* 3232A>G mutation detection using tgRNA (3232-1). *BRCA1* wild-type or 3232A>G dsDNAs are diluted in human plasma with a final concentration of 10^−18^ M. Error bars indicate s.e.m., *n* = 3. **P < 0.01; ****P < 0.0001; ns, no significance

To further expand the applications of CDetection in accurate diagnostics, we designed experiments using three targeting sgRNA and corresponding dsDNA activators (on- versus off-activator) to identify six common human ABO alleles [9] (Additional file 2: Table S1). Theoretically, CDetection carrying each of the three sgRNAs can identify O01, O02/O03, and B101, respectively. And if no fluorescent signal can be detected for all sgRNAs, the allele should be A101/A201 (Additional file 1: Figure S7a). Our results showed that CDetection failed to distinguish different ABO alleles as it produced indistinguishable fluorescent signals between the on- and off-dsDNA activator groups (Additional file 1: Figure S7b). To improve the specificity of CDetection, we introduced tuned guide RNA (tgRNA) containing a single-nucleotide mismatch in the spacer sequence [10], which transforms the undistinguishable state of two similar targets differed by a single base into the distinguishable state (Additional file 1: Figure S7c). To elucidate the feasibility of this enhanced CDetection, we repeated the ABO blood genotyping test. As our results indicated, CDetection with tgRNAs could determine the blood types with high accuracy in an antigen-antibody-independent and blood-free manner (Fig. 2b and Additional file 1: Figure S6c).

Disease-associated point mutations were usually detected by sequencing and probe detection [11]. However, sequencing is costly and time-consuming, and its sensitivity is dependent on sequencing depth, while probe-based methods perform poor sensitivity, particularly for single-nucleotide variation [11]. We sought to use CDetection to detect single-base mutations in the human genome. We selected the cancer-related *TP53* 856G>A mutation to test the feasibility. The results showed that CDetection could accurately distinguish the point mutated allele from the wild-type allele using selected tgRNAs (Additional file 1: Figure S8a and Additional file 2: Table S1). Furthermore, we applied CDetection platform in detecting two hotspots in breast cancer-related *BRCA1* gene (3232A>G and 3537A>G) [12]. CDetection with selected tgRNAs (tgRNA-3232-1 and tgRNA-3537-4) performed excellently to discriminate point mutations while sgRNAs could hardly support point mutation detection (Fig. 2c, d, Additional file 1: Figure S8b, c and Additional file 2: Table S1). As a proof of principle, we aimed to detect the human *BRCA1* gene, which contains a SNP (3232A>G) responsible for breast cancer [12]. We amplified the *BRCA1* wild-type allele and 3232A>G allele from DNAs in human breast cancer cell lines MDA-MB-468 (ATCC® HTB-132™) and SUM1315MO2 (BioIVT) respectively, using the RPA amplification approach. And tgRNA-programmed CDetection achieved to discriminate the SNP with strong fluorescent signal for 3232A>G SNP while with near-background signal for the wild-type allele (Fig. 2d and Additional file 1: Figure S8d) with allelic fractions as low as 1% (Additional file 1: Figure S8e). Meanwhile, the results also indicated that the targeted genomic DNAs without pre-amplification are not able to unleash the *trans*-cleavage activity of Cas12b enzyme (Additional file 1: Figure S8d). And the reason is possibly that Cas12b-RNA remains tightly bound to the cleave genomic DNAs, thus failing to enable the ssDNA substrate access and turnover [4, 13]. Furthermore, to mock the early clinical detection of primary diseases using cfDNA by CDetection, we diluted *BRCA1* 3232A>G dsDNAs into human plasma. Our results demonstrated that CDetection could achieve point mutation detection at the single-base resolution (Fig. 2e and Additional file 1: Figure S8f). These results demonstrate that CDetection with tgRNAs is able to achieve rapid DNA detection at the single-base resolution in clinical research.

The intrinsic features of base-pairing between spacer sequences and the corresponding target sequences make CRISPR-Cas systems exist potential off-target effect as previous works indicated [7, 14–22]. So we assessed the possible false positive of CDetection platform using potential off-targets containing 1 to 3 mismatches in their protospacer sequences predicted from targeting sgRNAs or tgRNAs using Cas-OFFinder [23] (Additional file 2: Table S1). The results showed that both sgRNA and tgRNA exhibited mismatch tolerance pattern (Additional file 1: Figure S9a, b) consistent with above results (Fig. 1a, b) and previous studies [4]. To eliminate the off-target-associated undesired false positive of CDetection, we indicated that a specific RPA pre-amplification was necessary (Additional file 1: Figure S9c, d).

Taken together, we establish a CDetection method based on the non-canonical ssDNA *trans*-cleavage properties of the Cas12b nuclease triggered by targeted dsDNA, which enables DNA detection with sub-attomolar sensitivity. The CDetection strategy can perform better than the Cas12a-DETECTR in sensitivity assay. Meanwhile, the CDetection is able to detect dsDNA directly without an extra step requiring in Cas13-SHERLOCK or Cas14-DETECTR platform [2, 3, 5, 6]. Moreover, we demonstrate that CDetection enables to achieve single-nucleotide sensitivity in DNA detection coupled with optimized tgRNA, which has been applied extendedly in other CRISPR-based detection platforms [2, 6, 24]. As a proof of concept, we use mock samples composed of diluted synthetic DNA and human plasma to demonstrate the capability of CDetection to perform direct cfDNA detection. However, the detection of clinical specimens may face bigger challenges due to the poor purity and integrity. We anticipate that CDetection will be used for the detection of cfDNA in clinical specimens in the future. Besides, the intrinsic features of CRISPR-Cas systems possessing mismatch tolerance [7, 14–22] and our results indicate that RPA pre-amplification process is necessary to produce strong signal and avoid false positive for CRISPR-Cas-based nucleic acid detection platforms. During the preparation process of this manuscript, an independent study reported a less sensitive nucleic acid detection platform [25] using previously reported AacCas12b protein [4] with loop-mediated isothermal amplification (LAMP). In their works, the authors generate ssDNA using asymmetric PCR or dsDNA using LAMP as an activator to perform SNP discrimination in the tested site [25], which is different from our strategy in principle. Our work, together with the AacCas12b-based work [25], demonstrates that Cas12b-based nucleic acid detection method should be a feasible platform for molecular diagnostics. This rapid and price-competitive DNA detection platform may have wide applications in molecular diagnostics and clinical research (Additional file 1: Figure S10). For instance, we estimate that over 20,000 known human disease-associated point mutations could be detected by CDetection (Additional file 1: Figure S11).

## Methods

### Protein purification

SpCas9 and LbCas12a proteins were commercially purchased (NEB). AaCas12b, ArCas12a, HkCas12a, and PrCas12a proteins were purified according to our previous report [7]. Briefly, BPK2014-Cas12-His_10_ plasmid was transformed into *E. coli* strain BL21 (λDE3) and protein expression was induced with 0.5 mM IPTG at 16 °C for 16 h. Cell pellets were harvested and lysed, followed by washing and elution using His60 Ni Superflow Resin (Takara). Purified Cas12 proteins were dialyzed, concentrated, and finally quantitated using the BCA Protein Assay Kit (Thermo Fisher).

### Nucleic acid preparation

DNA oligos were commercially purchased (Genscript). Double-stranded DNA activators were obtained by PCR reaction and purified using Oligo Clean & Concentrator Kit (ZYMO Research). In order to avoid false positive results caused by target strand (TS) ssDNA, we took non-target strand (NTS) ssDNA as PCR template. PCR primers and ssDNA templates were listed in Additional file 2: Table S1.

Guide RNAs (gRNAs: sgRNAs or crRNAs) were in vitro transcribed using HiScribe™ T7 High Yield RNA Synthesis Kit (NEB) and purified using MicroElute RNA Clean Up Kit (Omega). Targeting gRNAs containing a T7 promoter were used as transcription template. AaCas12b sgRNA (AasgRNA) and SpCas9 sgRNA templates for in vitro RNA transcription were PCR amplified using primers bearing a T7 promoter (Additional file 2: Table S1). Cas12a crRNA templates were obtained by annealing oligos containing a T7 promoter (Additional file 2: Table S1).

Background genomic DNAs used in indicated reactions were crudely purified from human embryonic kidney 293T cells using Mouse Direct PCR Kit (Bimake). To mimic the cell-free DNA (cfDNA), dsDNAs were diluted into human plasma (Thermo Fisher) at indicated concentrations.

### Fluorophore quencher (FQ)-labeled reporter assays

Detection assays were performed with 30 nM Cas12, 36 nM gRNA, 40 nM activator (unless otherwise indicated) mixed in 40 ng background genomic DNAs (in indicated reaction), 200 nM custom synthesized homopolymer ssDNA FQ reporter (Additional file 2: Table S1) and NEBuffer™ 2 (unless otherwise indicated) in a 20-μl reaction in a Corning® 384-well Polystyrene NBS Microplate. Reactions were incubated at 37 °C for indicated timecourse in a fluorescence plate reader (BioTek Synergy 4) with fluorescent kinetics measured every 5 min (*λ*_ex_ = 485 nm; *λ*_em_ = 528 nm, transmission gain = 61). The fluorescence results were analyzed by SigmaPlot software.

### Recombinase polymerase amplification (RPA) reactions

Recombinase polymerase amplification (RPA) reactions were proceeded using TwistAmp Basic (TwistDx) according to the manufacturer’s protocol. The 50-μl RPA reaction system containing varying amounts of DNA input was incubated in 37 °C for 10 min. Sixteen microliters of RPA product was directly transferred to the 20-μl detection assay as above mentioned.

### Statistical analysis

All the replicate experiments in this study consisted of three repeats. Uncertainties in the reported mean values are indicated as standard errors of the mean (s.e.m.). Statistical analysis was performed in SigmaPlot (version 12.0).

## Additional files

Additional file 1: **Figure S1.** Non-specific DNase activity is conserved across Cas12 proteins. **Figure S2.** The RuvC domain is responsible for ssDNA trans-cleavage. **Figure S3.** Preference for Cas12b-mediated trans-activated cleavage of non-specific ssDNA. **Figure S4.** Sensitivity and specificity of AaCas12b-mediated DNA detection. **Figure S5.** Comparison of sensitivity of AaCas12b- and LbCas12a-based DNA detection. **Figure S6.** CDetection achieves sub-attomolar sensitivity in DNA detection. **Figure S7.** Develop CDetection platform. **Figure S8.** Accurate DNA detection using CDetection platform. **Figure S9.** Potential off-target analysis of CDetection. **Figure S10.** Rapid and accurate diagnostic applications of CDetection. **Figure S11.** Genetic variants from ClinVar that, in principle, can be detected by CDetection platform. (PDF 2218 kb)

Additional file 2: **Table S1.** Nucleic acids used in this study. **Table S2.** The components of buffers tested for Cas12b-mediated DNA detection in this study. (PDF 293 kb)

Additional file 3: Review history. (DOCX 1323 kb)

## Data Availability

All supporting data are included in the manuscript and supplemental files. AaCas12b/AaC2c1 expression plasmid is available from Addgene under a Uniform Biological Material Transfer Agreement.
