# Supplementary material for: CDetection: CRISPR-Cas12b-based DNA detection with sub-attomolar sensitivity and single-base specificity
Source: Genome Biol. 2019 Jul 1;20:132. doi: 10.1186/s13059-019-1742-z (PMC6604390; doi:10.1186/s13059-019-1742-z)
Supplement: Supplementary file 1 — Figure S1. Non-specific DNase activity is conserved across Cas12 proteins. Figure S2. The RuvC domain is responsible for ssDNA trans-cleavage. Figure S3. Preference for Cas12b-mediated trans-activated cleavage of non-specific ssDNA. Figure S4. Sensitivity and specificity of AaCas12b-mediated DNA detection. Figure S5. Comparison of sensitivity of AaCas12b- and LbCas12a-based DNA detection. Figure S6. CDetection achieves sub-attomolar sensitivity in DNA detection. Figure S7. Develop CDetection platform. Figure S8. Accurate DNA detection using CDetection platform. Figure S9. Potential off-target analysis of CDetection. Figure S10. Rapid and accurate diagnostic applications of CDetection. Figure S11. Genetic variants from ClinVar that, in principle, can be detected by CDetection platform. (PDF 2218 kb) [file 13059_2019_1742_MOESM1_ESM.pdf]

Figure S1

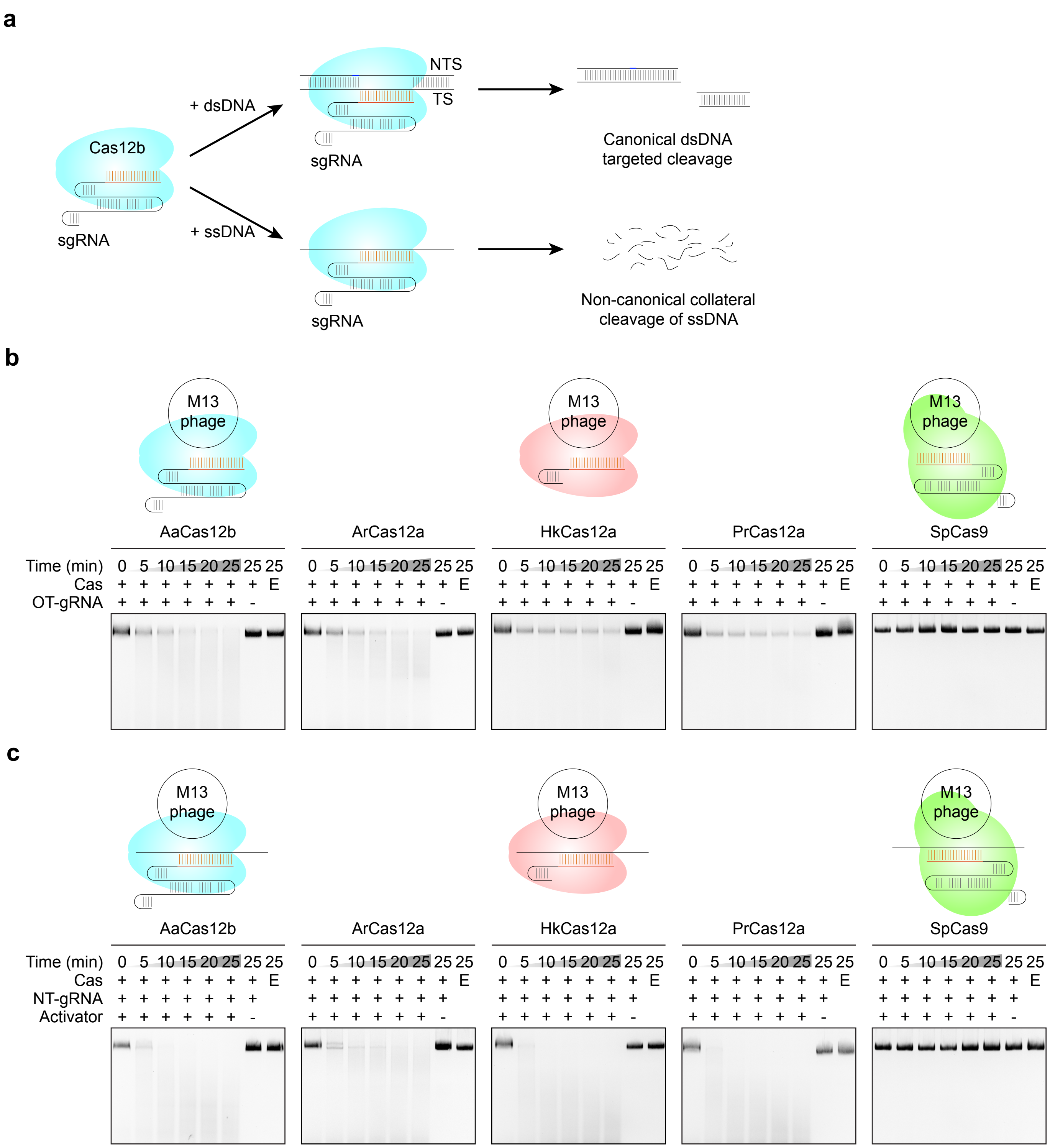
a
Cas12b
sgRNA
+ dsDNA
+ ssDNA
NTS
TS
sgRNA
sgRNA
Canonical dsDNA targeted cleavage
Non-canonical collateral cleavage of ssDNA
b
M13 phage
AaCas12b
ArCas12a
HkCas12a
PrCas12a
SpCas9
Time (min)
Cas
OT-gRNA
0 5 10 15 20 25 25 25
+ + + + + + + E
+ + + + + + -
c
M13 phage
AaCas12b
ArCas12a
HkCas12a
PrCas12a
SpCas9
Time (min)
Cas
NT-gRNA
Activator
0 5 10 15 20 25 25 25
+ + + + + + + E
+ + + + + + +
+ + + + + + -

Figure S1 Non-specific DNase activity is conserved across Cas12 proteins. a Schematics illustrating Cas12b possesses the ability of canonical target recognition and cleavage of dsDNA, as well as non-canonical collateral cleavage of ssDNA. **b** M13mp18 ssDNA cleavage timecourses with purified AaCas12b, ArCas12a, HkCas12a, PrCas12a and SpCas9 coupled with an on-target guide RNA (OT-gRNA) complementary to M13 phage. c M13mp18 ssDNA cleavage timecourses with purified AaCas12b, ArCas12a, HkCas12a, PrCas12a and SpCas9 coupled with a non-target guide RNA (NT-gRNA) and complementary ssDNA activator with no sequence homology to M13 phage.

Figure S2

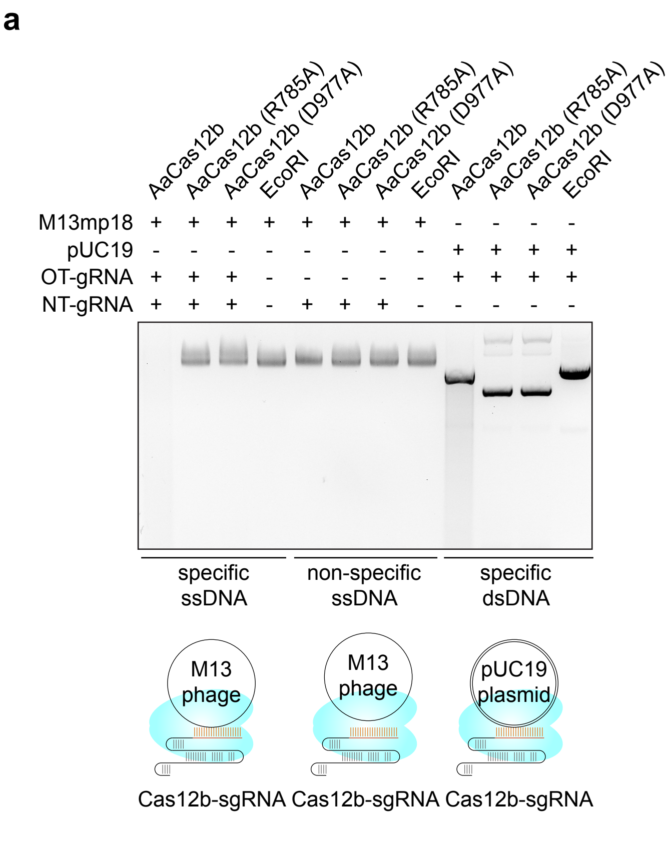
a
AaCas12b
AaCas12b (R785A)
AaCas12b (D977A)
EcoRI
M13mp18 + + + + + + + + - - - -
pUC19 - - - - - - - - + + + +
OT-gRNA + + + - - - - - + + + +
NT-gRNA + + + - + + + - - - - -
specific ssDNA
non-specific ssDNA
specific dsDNA
M13 phage
pUC19 plasmid
Cas12b-sgRNA
Cas12b-sgRNA
Cas12b-sgRNA

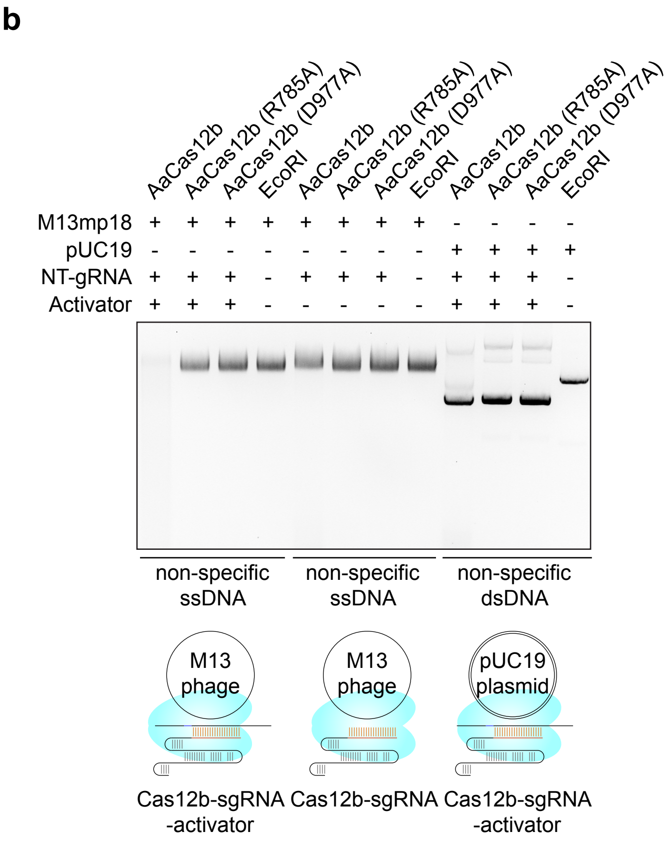
b
AaCas12b
AaCas12b (R785A)
AaCas12b (D977A)
EcoRI
M13mp18 + + + + + + + + - - - -
pUC19 - - - - - - - - + + + +
NT-gRNA + + + - + + + - + + + -
Activator + + + - - - - - + + + -
non-specific ssDNA
non-specific ssDNA
non-specific dsDNA
M13 phage
pUC19 plasmid
Cas12b-sgRNA -activator
Cas12b-sgRNA
Cas12b-sgRNA -activator

Figure S2 The RuvC domain is responsible for ssDNA trans-cleavage. a-b M13mp18 ssDNA substrate and pUC19 dsDNA cleavage timecourses with purified WT AaCas12b and RuvC catalytic mutants coupled with **an** on-target gRNA (OT-gRNA) or non-target gRNA (NT-gRNA) or **b** a NT-gRNA and complementary ssDNA activator with no sequence homology to M13 phage or pUC19.

Figure S3

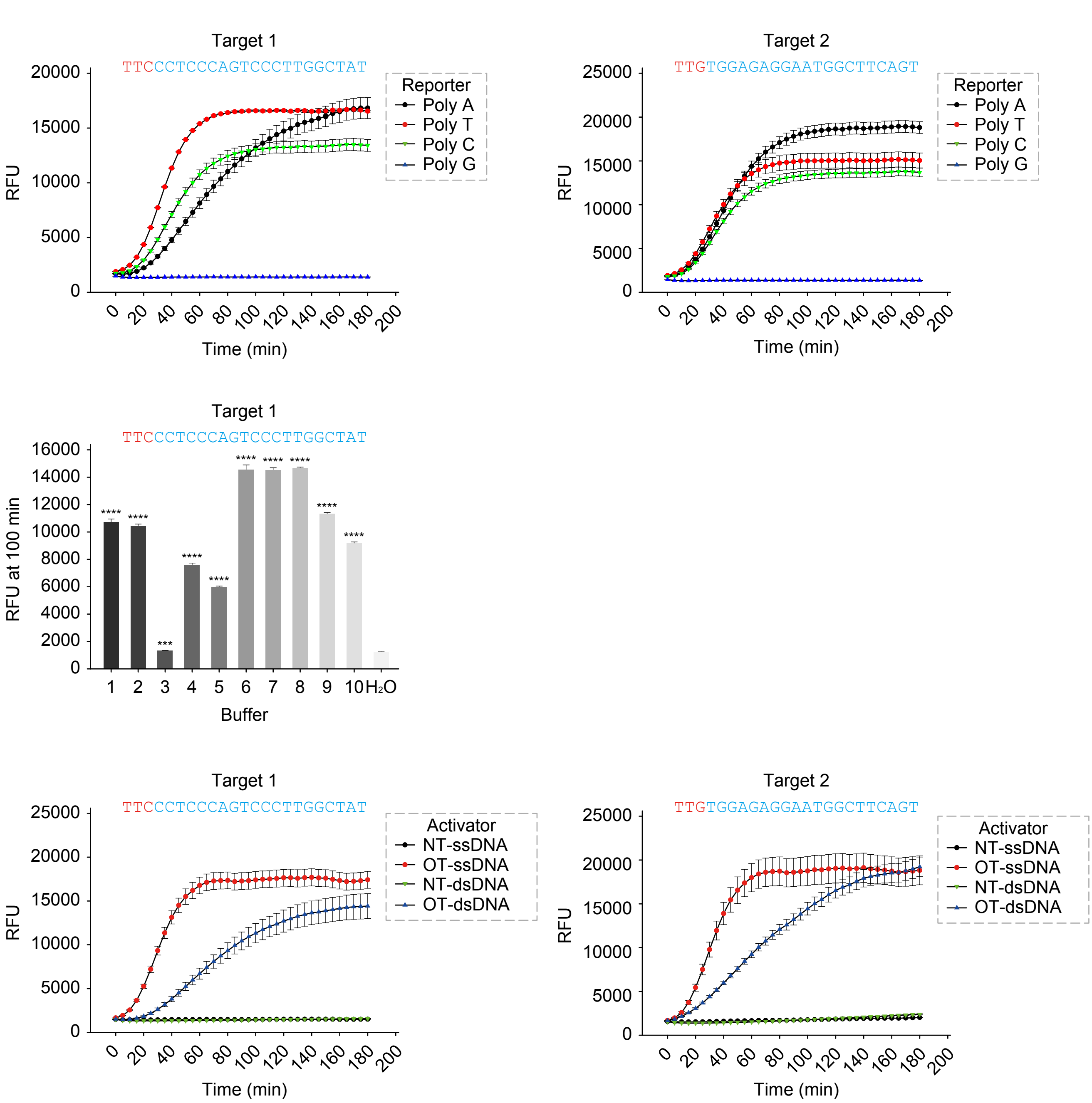

a
Target 1
TTCCCTCCCAGTCCCTTGGCTAT
RFU
Time (min)
Reporter
Poly A
Poly T
Poly C
Poly G
Target 2
TTGTGGAGAGGAATGGCTTCAGT
b
Target 1
TTCCCTCCCAGTCCCTTGGCTAT
RFU at 100 min
Buffer
1 2 3 4 5 6 7 8 9 10 H2O
c
Target 1
TTCCCTCCCAGTCCCTTGGCTAT
Activator
NT-ssDNA
OT-ssDNA
NT-dsDNA
OT-dsDNA
Target 2
TTGTGGAGAGGAATGGCTTCAGT

Figure S3 Preference for Cas12b-mediated *trans*-activated cleavage of non-specific ssDNA. **a** Base preference of AaCas12b-mediated *trans*-cleavage of ssDNA reporters containing a homoploymer of A, T, G or C bases. AaCas12b is incubated with a sgRNA targeting a synthetic ssDNA 1 or 2. PAM sequences are colored in red, protospacers are colored in blue. Error bars indicate standard errors of the mean (s.e.m.), n = 3. RFU, relative fluorescence units. **b** A variety of buffers are tested for their effect on AaCas12b-mediated trans-cleavage activity. AaCas12b is incubated with a sgRNA targeting a synthetic dsDNA 1. Error bars indicate (s.e.m.), n = 3. Two-tailed Student's *t* test is used for significance analysis between test group and the control group (H_2O), respectively. ***P < 0.001; ****P < 0.0001. **c** *Trans*-cleavage activity analysis for AaCas12b using on-target-ssDNA (OT-ssDNA), non-target-ssDNA (NT-ssDNA), on-target-ssDNA (OT-dsDNA) and non-target-dsDNA (NT-dsDNA) as activator. AaCas12b is incubated with a sgRNA targeting a synthetic ssDNA 1 or 2. Error bars indicate s.e.m., n = 3.

Figure S4

a

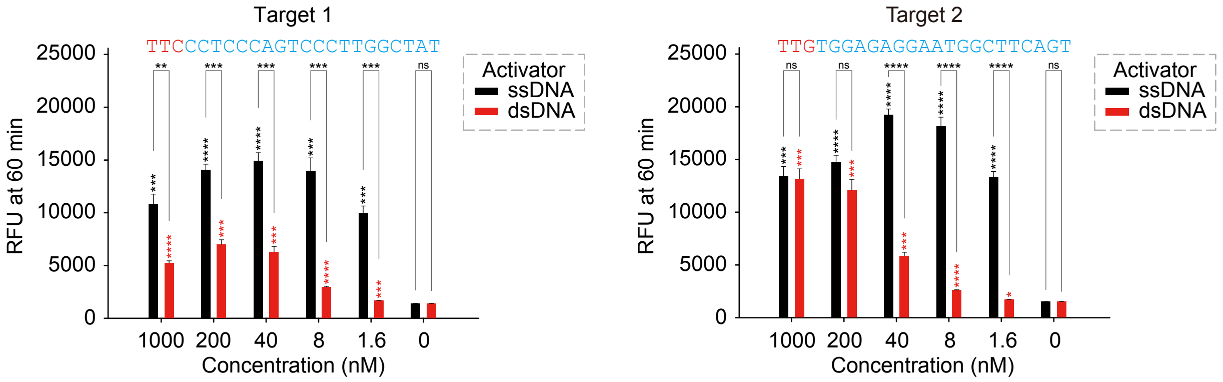

b

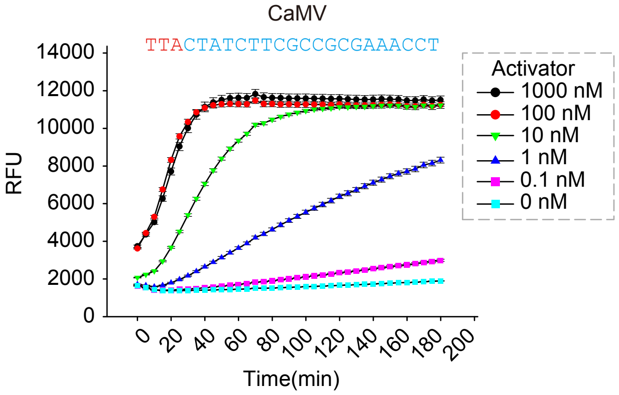

c

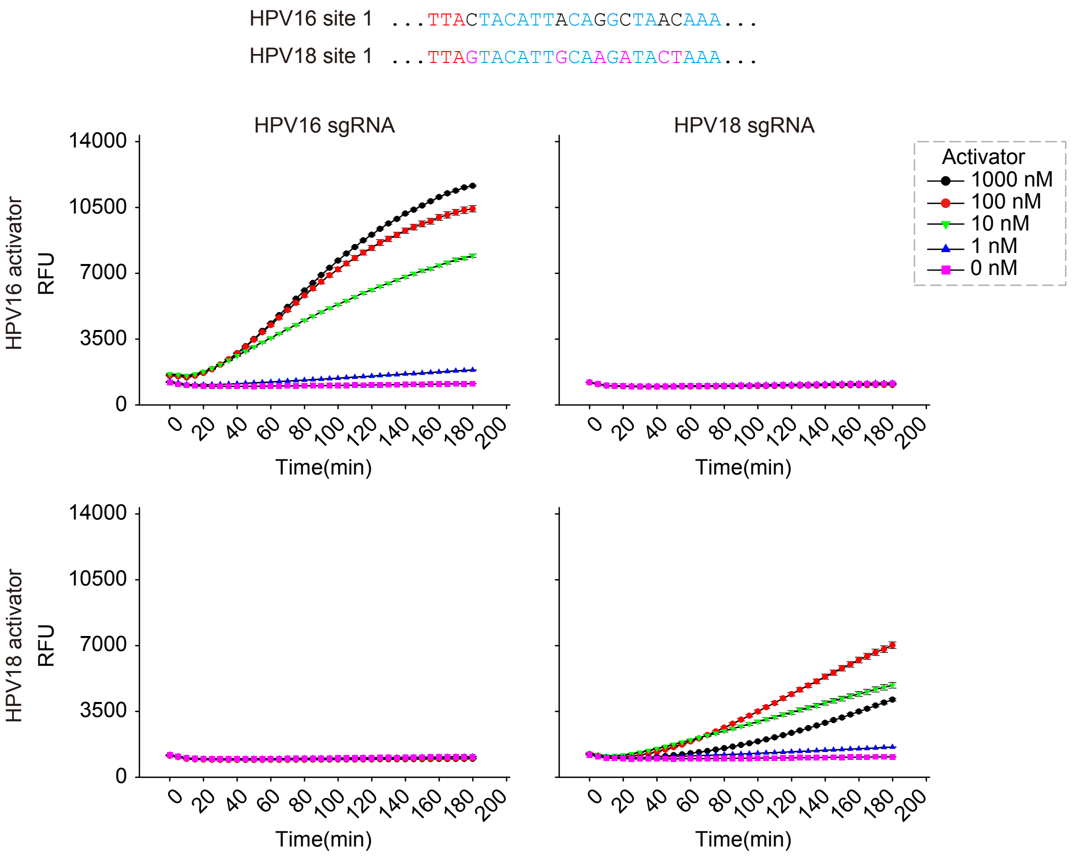

d

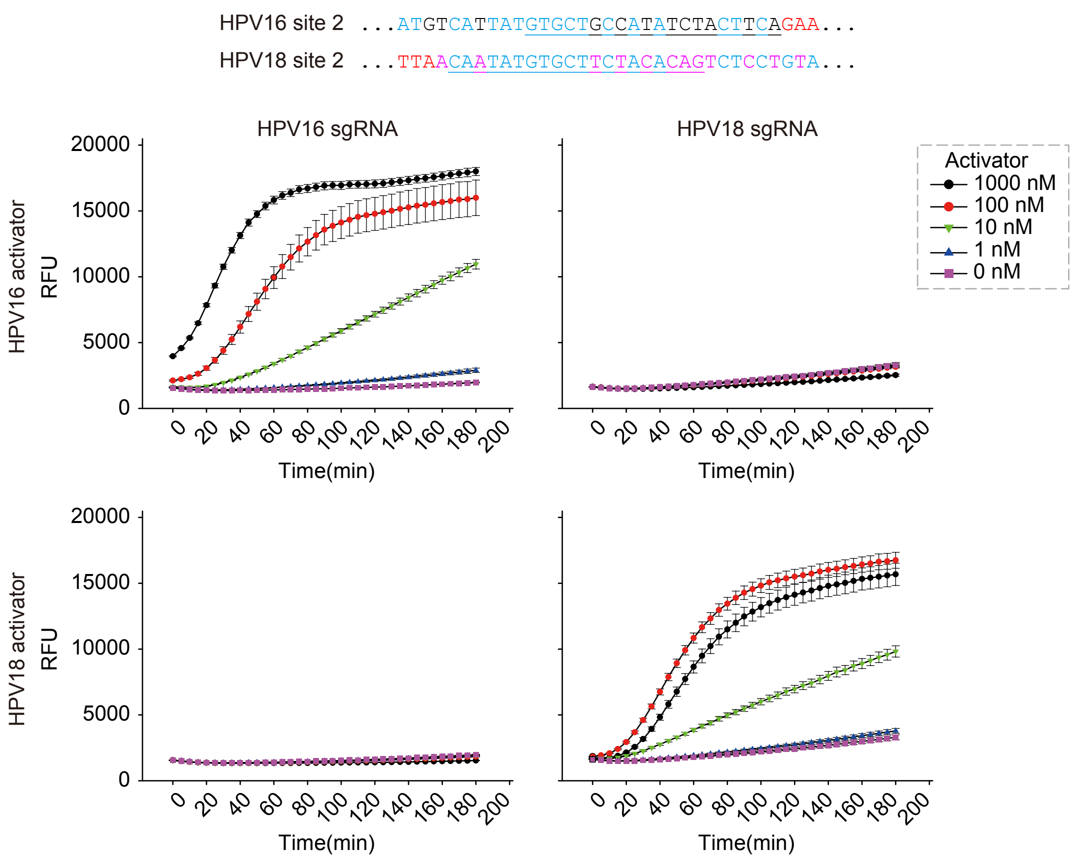

HPV16 site 2 ...ATGTCATTATGTGCTGCCATATCTACTTCAGAA...
HPV18 site 2 ...TTAACAATATGTGCTTCTACACAGTCTCCTGTA...
HPV16 sgRNA
HPV18 sgRNA
HPV16 activator
HPV18 activator
RFU
20000
15000
10000
5000
0
0 20 40 60 80 100 120 140 160 180 200
Time(min)
Activator
1000 nM
100 nM
10 nM
1 nM
0 nM

e

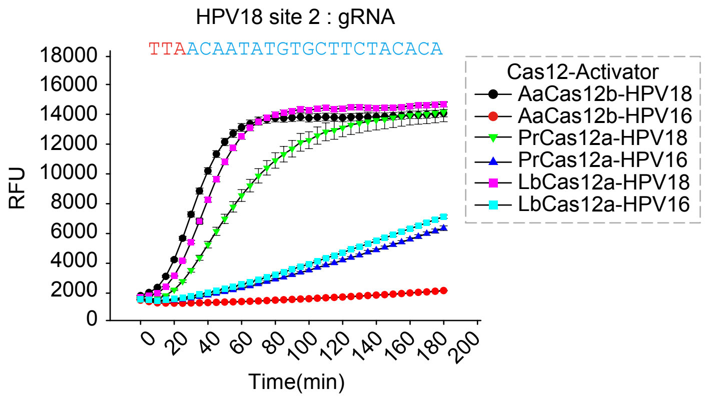

HPV18 site 2 : gRNA
TTAACAATATGTGCTTCTACACA
RFU
18000
16000
14000
12000
10000
8000
6000
4000
2000
0
0 20 40 60 80 100 120 140 160 180 200
Time(min)
Cas12-Activator
AaCas12b-HPV18
AaCas12b-HPV16
PrCas12a-HPV18
PrCas12a-HPV16
LbCas12a-HPV18
LbCas12a-HPV16

f

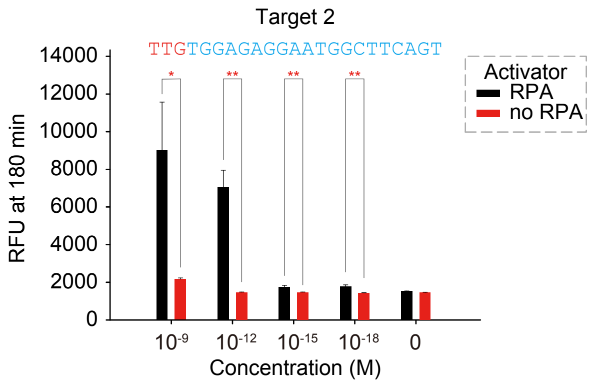

Target 2
TTGTGGAGAGGAATGGCTTCAGT
RFU at 180 min
14000
12000
10000
8000
6000
4000
2000
0
*
**
**
**
10-9
10-12
10-15
10-18
0
Concentration (M)
Activator
RPA
no RPA

Figure S4 Sensitivity and specificity of AaCas12b-mediated DNA detection. a Comparison of *trans*-cleavage activity for AaCas12b using ssDNA or dsDNA activator without pre-amplification. AaCas12b is incubated with a sgRNA targeting a synthetic ssDNA or dsDNA. PAM sequences are colored in red, protospacers are colored in blue, SNPs are colored in pink. Error bars indicate standard errors of the mean (s.e.m.), n = 3. RFU, relative fluorescence units. Two-tailed Student's *t* test is used for significance analysis between test group and the control group (0 nM), respectively. **b** Fluorescence timecourses showing AaCas12b-sgRNA detects the existence of CaMV DNA. Error bars indicate (s.e.m.), n = 3. **c-d** (Upper) Schematics showing HPV16 and HPV18 sequences targeted by AaCas12b. Red highlighted bases indicate 5'PAM sequences; Blue and green highlighted bases indicate different nucleotides. (*Lower*) AaCas12b distinguishes two synthetic HPV sequences (HPV16 and HPV18) at **c** site 1 and **d** site 2. Error bars indicate (s.e.m.), n = 3. **e** Comparison of the specificity among AaCas12b, PrCas12a and LbCas12a in dsDNA distinguishability using synthetic HPV18 activator (site 2). Error bars indicate (s.e.m.), n = 3. **f** Comparison of *trans*-cleavage activity and pre-amplification enhanced *trans*-cleavage activity for AaCas12b using dsDNA activator. AaCas12b is incubated with a sgRNA targeting a synthetic ssDNA 2 or dsDNA 2. Error bars indicate (s.e.m.), n = 3. Two-tailed Student's *t* test is used for significance analysis between the two samples in group. *$P < 0.05$; **$P < 0.01$; ***$P < 0.001$; ****$P < 0.0001$.

Figure S5

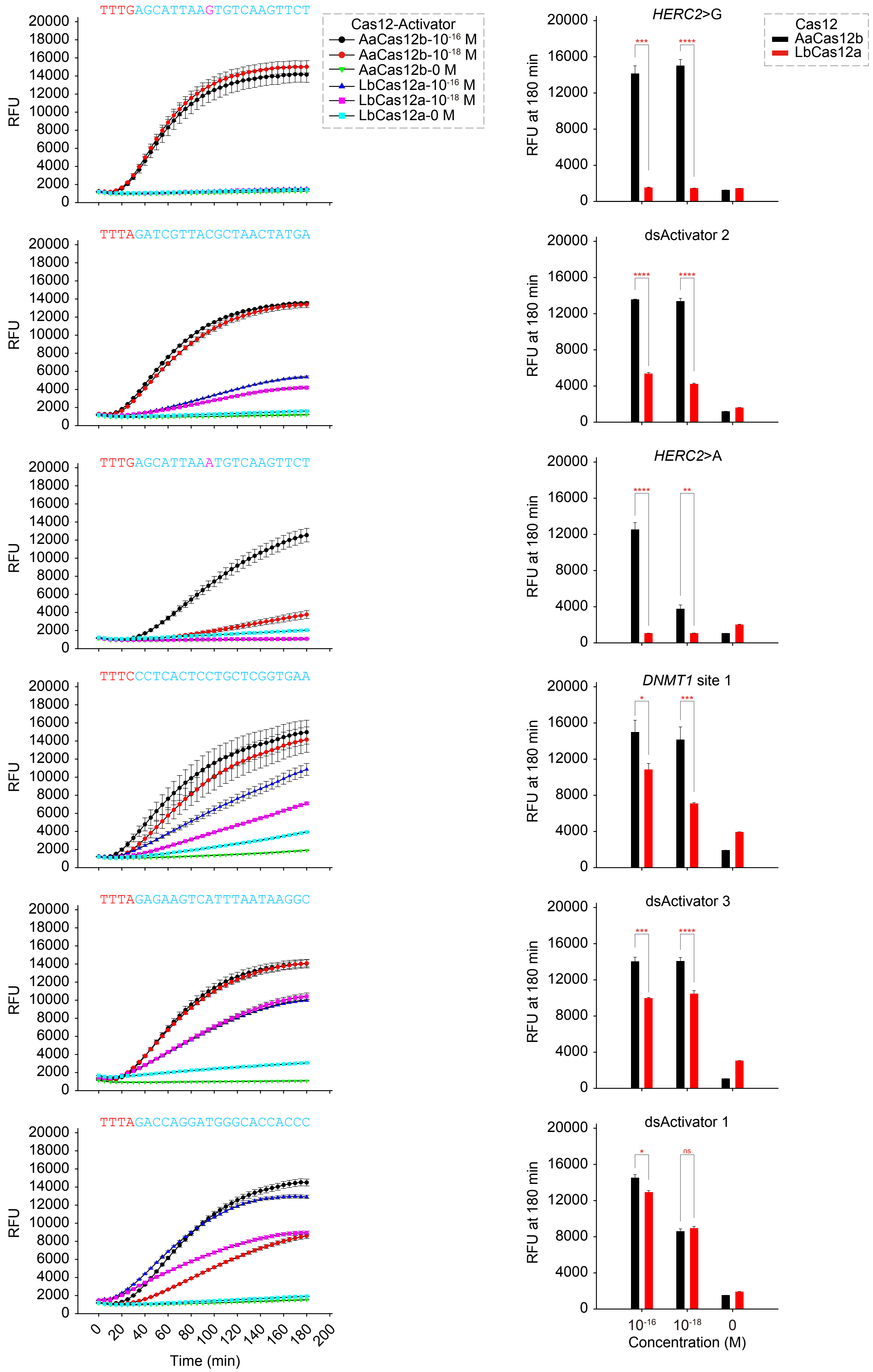
TTTGAGCATTAAGTGTCAAGTTCT
TTTAGATCGTTACGCTAACTATGA
TTTGAGCATTAAATGTCAAGTTCT
TTTCCCTCACTCCTGCTCGGTGAA
TTTAGAGAAGTCATTTAATAAGGC
TTTAGACCAGGATGGGCACCACCC
Cas12-Activator
AaCas12b-10-16 M
AaCas12b-10-18 M
AaCas12b-0 M
LbCas12a-10-16 M
LbCas12a-10-18 M
LbCas12a-0 M
RFU
Time (min)
HERC2>G
dsActivator 2
HERC2>A
DNMT1 site 1
dsActivator 3
dsActivator 1
RFU at 180 min
Cas12
AaCas12b
LbCas12a
ns
10-16
10-18
0
Concentration (M)

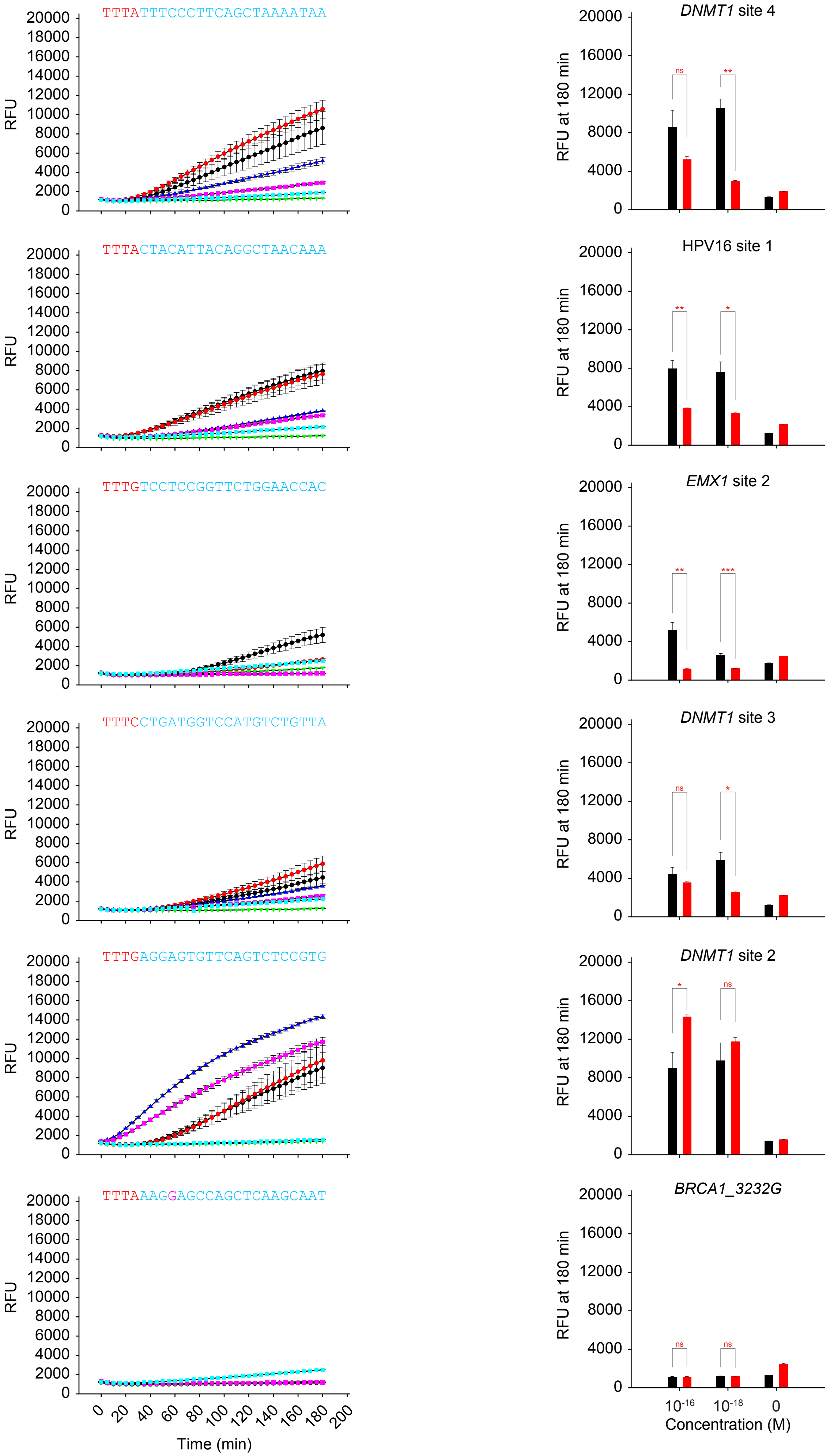
TTTATTTCCCTTCAGCTAAAATAA
TTTACTACATTACAGGCTAACAAA
TTTGTCCTCCGGTTCTGGAACCAC
TTTCCTGATGGTCCATGTCTGTTA
TTTGAGGAGTGTTCAGTCTCCGTG
TTTAAAGGAGCCAGCTCAAGCAAT
RFU
Time (min)
DNMT1 site 4
HPV16 site 1
EMX1 site 2
DNMT1 site 3
DNMT1 site 2
BRCA1_3232G
RFU at 180 min
10^{-16}
10^{-18}
0
Concentration (M)

Figure S5 Comparison of sensitivities of AaCas12b- and LbCas12a-based DNA detection. (*Left*) Fluorescence timecourses obtained from AaCas12b- and LbCas12a-based DNA detection with RPA pre-amplification. Cas12 proteins are incubated with their cognate gRNAs targeting synthetic dsDNAs with a final concentration of 10^{-16} M and 10^{-18} M. PAM sequences are colored in red, protospacers are colored in blue, SNPs are colored in pink. Error bars indicate standard errors of the mean s.e.m., n = 3. (*Right*) Maximum fluorescence signal obtained from timecourses in the left panel. Error bars indicate (s.e.m.), n = 3. Two-tailed Student's *t* test is used for significance analysis between the two samples in group. $*P < 0.05$; $**P < 0.01$; $***P < 0.001$; $****P < 0.0001$; ns, no significance.

Figure S6

a

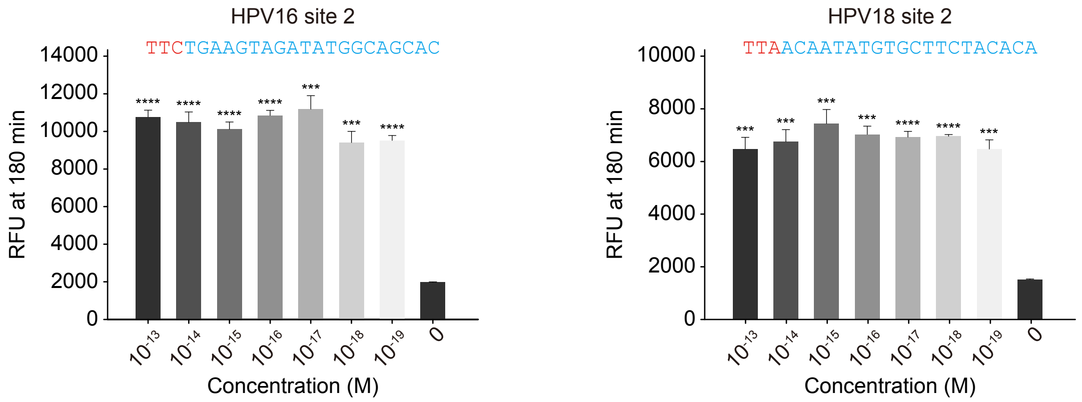

HPV16 site 2
TTCTGAAGTAGATATGGCAGCAC
RFU at 180 min
Concentration (M)
HPV18 site 2
TTAACAATATGTGCTTCTACACA
RFU at 180 min
Concentration (M)

b

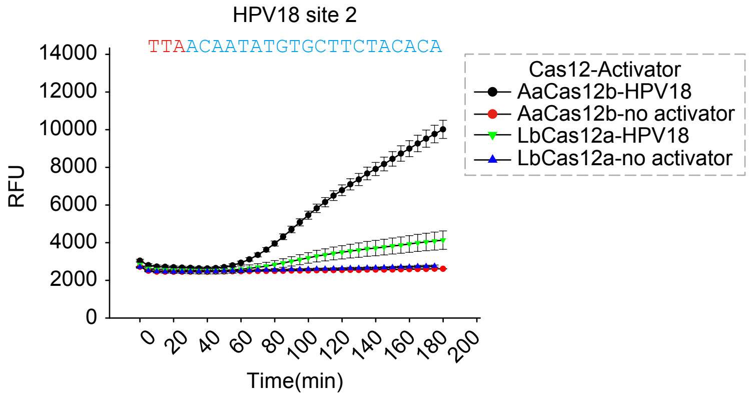

HPV18 site 2
TTAACAATATGTGCTTCTACACA
Cas12-Activator
AaCas12b-HPV18
AaCas12b-no activator
LbCas12a-HPV18
LbCas12a-no activator
RFU
Time(min)

Figure S6 CDetection achieves sub-attomolar sensitivity in DNA detection. a CDetection achieves sub-attomolar (0.1 åM) sensitivity in detection of HPV16 and HPV18 dsDNAs mixed with background genome. PAM sequences are colored in red, protospacers are colored in blue. Error bars indicate standard errors of the mean (s.e.m.), n = 3. RFU, relative fluorescence units. Two-tailed Student's *t* test is used for significance analysis between test group and the control group (0 nM), respectively. ***P < 0.001; ****P < 0.0001. **b** Fluorescence timecourses obtained from AaCas12b- and LbCas12a-based DNA detection with RPA pre-amplification. Cas12 is incubated with a cognate gRNA targeting a synthetic HPV18 dsDNA diluted in human plasma with a final concentration of 10^{-18} M. Error bars indicate (s.e.m.), n = 3.

Figure S7

a

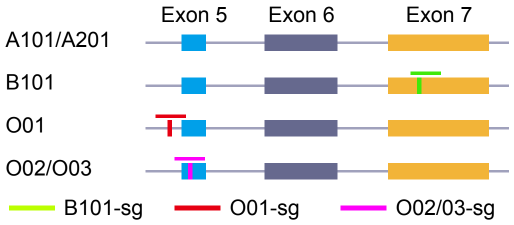

ABO	sgRNA B101	sgRNA O01	sgRNA O02/03
A101/A201	-	-	-
B101	+	-	-
O01	-	+	-
O02/O03	-	-	+

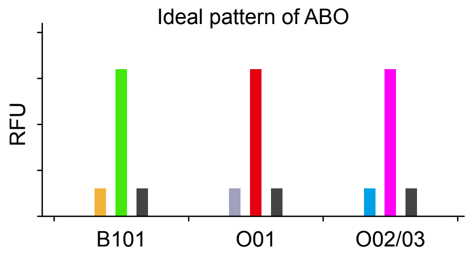

b

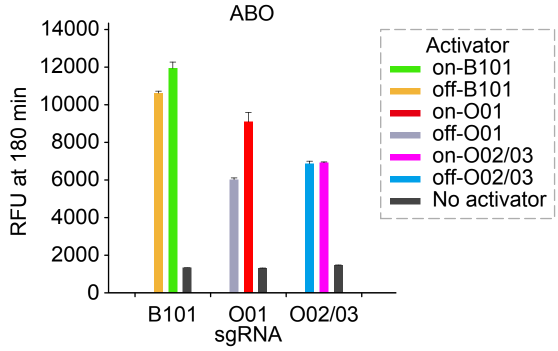

c

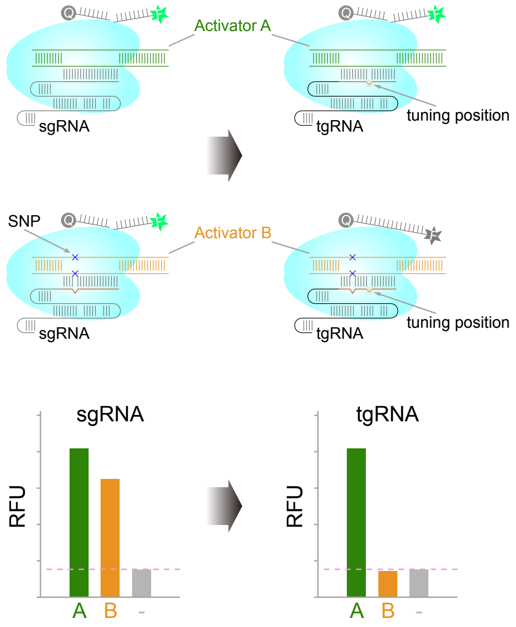

Figure S7 Develop CDetection platform. a (*Upper*) Schematics showing the ABO blood genotyping detected by CDetection. Six common ABO alleles and three targeting sgRNAs are shown. PAM sequences are colored in red, protospacers are colored in blue, SNPs are colored in pink, base substitution in tgRNAs are colored in orange. (*Lower*) Each sgRNA distinguishes an identical allele with detectable signal. If all three sgRNAs produce no signal, the allele is A101 or A201. **b** Fluorescence single obtained in ABO blood genotyping detection using CDetection. CDetection cannot distinguish two dsDNA activators differed by only one single-base polymorphism (on-B101 *vs*. off-B101, on-O01 *vs*. off-O01, on-O02/03 *vs*. off-O02/03). Error bars indicate standard errors of the mean (s.e.m.), n = 3. RFU, relative fluorescence units. **c** (*Upper*) Schematics showing the development of CDetection by introducing tuned gRNA (tgRNA). (*Lower*) CDetection using sgRNA cannot distinguish two dsDNA activators differed by a single mismatch, while CDetecion using tgRNA, which bears a mismatch within the spacer sequence, can achieve DNA detection with a single-base-resolution.

Figure S8

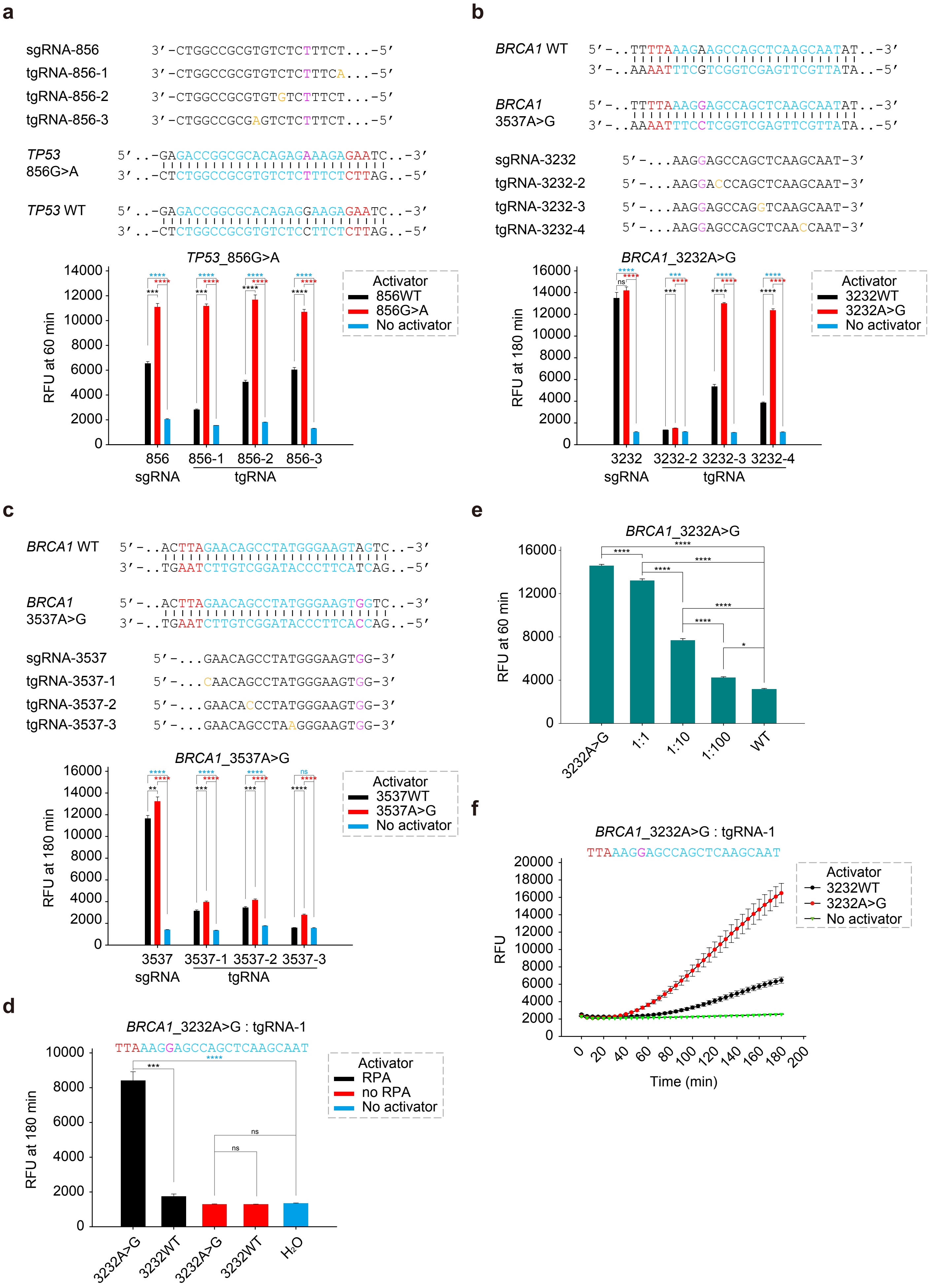

a
sgRNA-856 3′-CTGGCCGCGTGTCTCTTTCT...-5′
tgRNA-856-1 3′-CTGGCCGCGTGTCTCTTTCA...-5′
tgRNA-856-2 3′-CTGGCCGCGTGTGTCTTTCT...-5′
tgRNA-856-3 3′-CTGGCCGCGAGTCTCTTTCT...-5′
TP53 856G>A 5′..-GAGACCGGCGCACAGAGAAAGAGAATC..-3′
3′..-CTCTGGCCGCGTGTCTCTTTCTCTTAG..-5′
TP53 WT 5′..-GAGACCGGCGCACAGAGGAAGAGAATC..-3′
3′..-CTCTGGCCGCGTGTCTCCTTCTCTTAG..-5′
TP53_856G>A
RFU at 60 min
Activator
856WT
856G>A
No activator
856
sgRNA
856-1
856-2
856-3
tgRNA
b
BRCA1 WT 5′-..TTTTAAAGAAGCCAGCTCAAGCAATAT..-3′
3′-..AAAATTTCGTCGGTCGAGTTCGTTATA..-5′
BRCA1 3537A>G 5′-..TTTTAAAGGAGCCAGCTCAAGCAATAT..-3′
3′-..AAAATTTCCTCGGTCGAGTTCGTTATA..-5′
sgRNA-3232 5′-...AAGGAGCCAGCTCAAGCAAT-3′
tgRNA-3232-2 5′-...AAGGACCCAGCTCAAGCAAT-3′
tgRNA-3232-3 5′-...AAGGAGCCAGGTCAAGCAAT-3′
tgRNA-3232-4 5′-...AAGGAGCCAGCTCAACCAAT-3′
BRCA1_3232A>G
RFU at 180 min
Activator
3232WT
3232A>G
No activator
3232
sgRNA
3232-2
3232-3
3232-4
tgRNA
c
BRCA1 WT 5′-..ACTTAGAACAGCCTATGGGAAGTAGTC..-3′
3′-..TGAATCTTGTCGGATACCCTTCATCAG..-5′
BRCA1 3537A>G 5′-..ACTTAGAACAGCCTATGGGAAGTGGTC..-3′
3′-..TGAATCTTGTCGGATACCCTTCACCAG..-5′
sgRNA-3537 5′-...GAACAGCCTATGGGAAGTGG-3′
tgRNA-3537-1 5′-...CAACAGCCTATGGGAAGTGG-3′
tgRNA-3537-2 5′-...GAACACCCTATGGGAAGTGG-3′
tgRNA-3537-3 5′-...GAACAGCCTAAGGGAAGTGG-3′
BRCA1_3537A>G
RFU at 180 min
Activator
3537WT
3537A>G
No activator
3537
sgRNA
3537-1
3537-2
3537-3
tgRNA
d
BRCA1_3232A>G : tgRNA-1
TTAAAGGAGCCAGCTCAAGCAAT
RFU at 180 min
Activator
RPA
no RPA
No activator
3232A>G
3232WT
3232A>G
3232WT
H2O
e
BRCA1_3232A>G
RFU at 60 min
3232A>G
1:1
1:10
1:100
WT
f
BRCA1_3232A>G : tgRNA-1
TTAAAGGAGCCAGCTCAAGCAAT
RFU
Time (min)
Activator
3232WT
3232A>G
No activator

Figure S8 Accurate DNA detection using CDetection platform. a (*Upper*) Schematics showing the sequence variation within *TP53* gene along with targeting sgRNA and tgRNAs. PAM sequences are colored in red, protospacers are colored in blue, SNPs are colored in pink, base substitution in tgRNAs are colored in orange. (*Lower*) Maximum fluorescence signal showing the sensitivity for human *TP53* 856G>A mutation detection using sgRNA and tgRNAs. Error bars indicate standard errors of the mean (s.e.m.), n = 3. RFU, relative fluorescence units; tgRNA, tuned guide RNA. Two-tailed Student's *t* test is used for significance analysis among each other in group. **(b-c)** (*Upper*) Schematics showing the sequence variation within *BRCA1* gene along with targeting sgRNA and tgRNAs. (*Lower*) Maximum fluorescence signal showing the sensitivity for human *BRCA1* **b** 3232A>G and **c** 3537A>G mutation detection using sgRNA and tgRNAs. Error bars indicate (s.e.m.), n = 3. Two-tailed Student's *t* test is used for significance analysis among each other in group. **d** Maximum fluorescence signal obtained from timecourses in **Fig. 2d**. CDetection is conducted using RPA-amplified dsDNAs from genomic DNAs or genomic DNAs without RPA-amplification directly. *BRCA1* wild-type genomic DNAs or 3232A>G mutant genomic DNAs are extracted from cell lines. Error bars indicate (s.e.m.), n = 3. Two-tailed Student's *t* test is used for significance analysis among each other in group. **e** Maximum fluorescence signal showing the sensitivity for human *BRCA1*_3232A>G detection using selected tgRNA (*BRCA1*_3232A>G tgRNA-1). Mutant DNAs (*BRCA1*_3232A>G), wildtype DNAs (*BRCA1*_3232WT) and mixtures (mutant:WT = 1:1, 1:10 and 1:100) are used in CDetection analysis. *BRCA1* wild-type genomic DNAs or 3232A>G mutant genomic DNAs are extracted from cell lines. Error bars indicate standard errors of the mean (s.e.m.), n = 3. Two-tailed Student's *t* test is used for significance analysis among each other. **f** Fluorescence timecourses showing the sensitivity and specificity of CDetection with RPA for human *BRCA1* 3232A>G mutation detection using tgRNA (3232-1). Wild-type *BRCA1* or *BRCA1* 3232A>G dsDNAs are diluted in human plasma with a final concentration of 10^{-16} M. Error bars indicate (s.e.m.), n = 3. **$P < 0.01$; ***$P < 0.001$; ****$P < 0.0001$; ns, no significance.

Figure S9

a

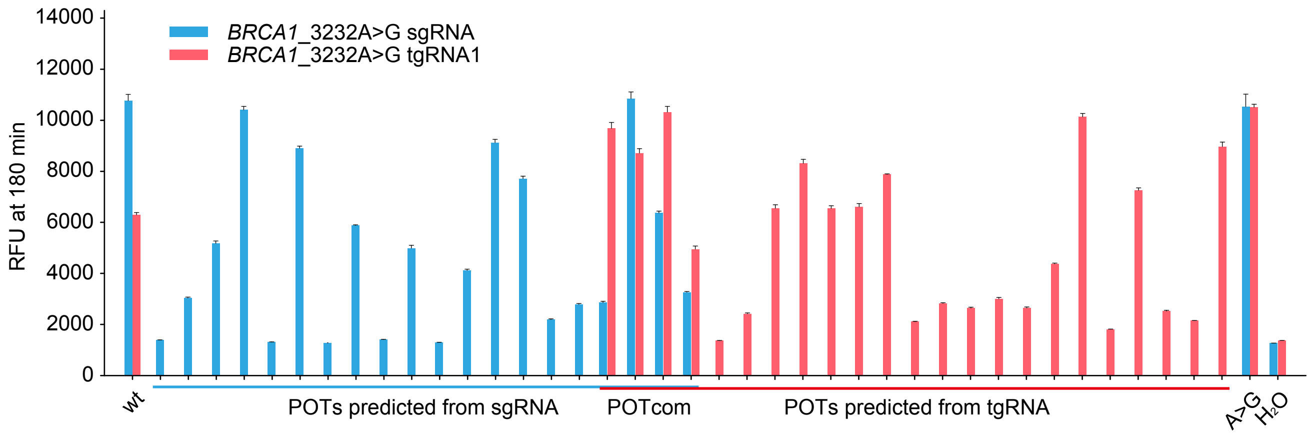

b

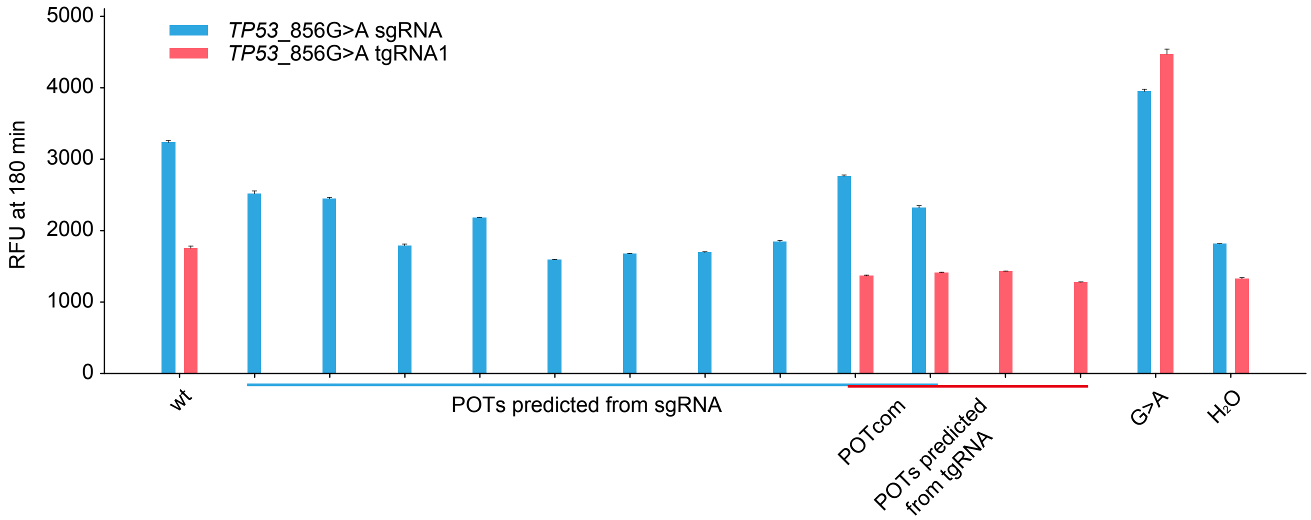

c

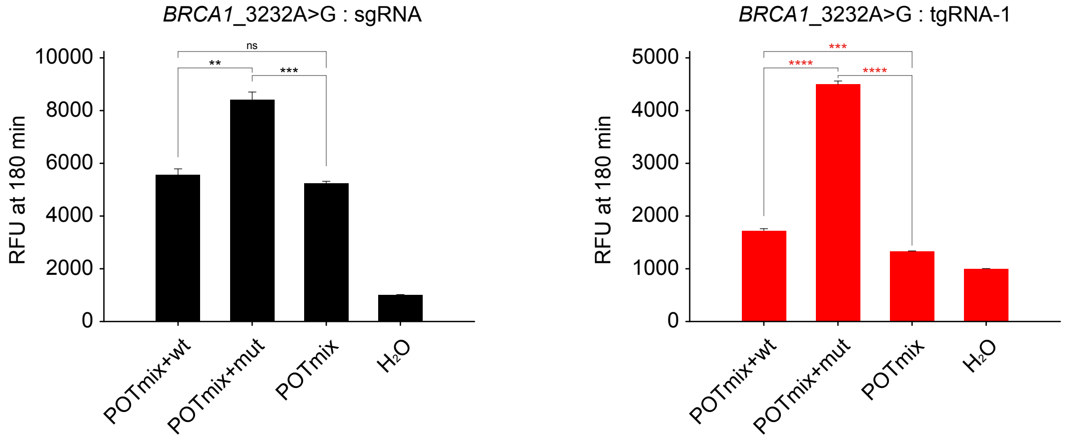

d

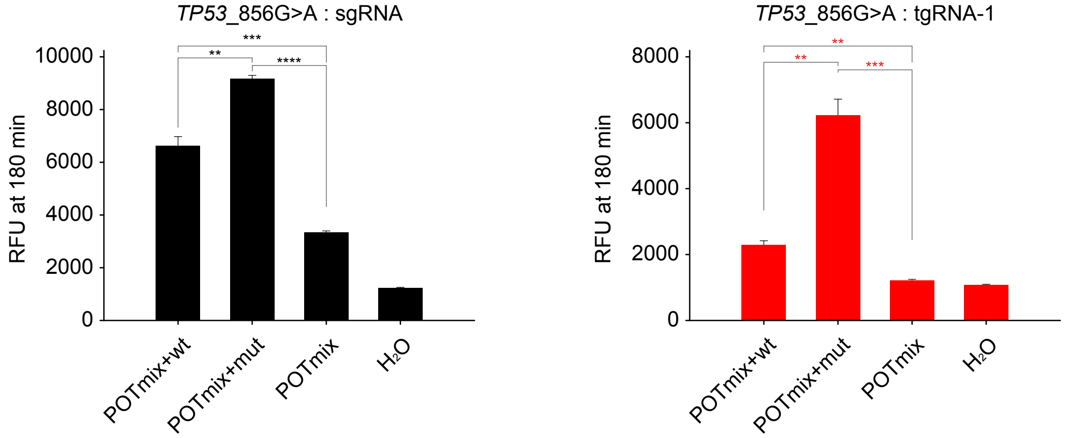

Figure S9 Potential off-target analysis of CDetection. a-b Maximum fluorescence signal obtained from sgRNA- and tgRNA-based CDetection using potential off-targets without RPA pre-amplification. AaCas12b is incubated with sgRNA or tgRNA targeting their cognate synthetic **a** *BRCA1* 3232A>G or **b** *TP53* 856 G>A off-target dsDNAs individually. Potential off-target sites bearing 1 to 3 mismatches are predicted using Cas-OFFinder. POT, potential off-target; POTcom, common POTs predicted from both sgRNA and tgRNA. Error bars indicate standard errors of the mean (s.e.m.), n = 3. **c-d** Maximum fluorescence signal obtained from sgRNA or tgRNA-based CDetection using mixed off-targets after RPA pre-amplification. All potential off-targets from sgRNA and tgRNA targeting **c** *BRCA1* 3232A<G or **d** *TP53* G>A are mixed together and amplified using RPA. POTmix, mixture of all potential off-targets of sgRNA and tgRNA. POTmix+wt, mixture of POTmix and wild-type on-target DNA substrate. POTmix+mut, mixture of POTmix and mutant on-target DNA substrate. Error bars indicate (s.e.m.), n = 3. Two-tailed Student's *t* test is used for significance analysis. **$P < 0.01$; ***$P < 0.001$; ****$P < 0.0001$; ns, no significance.

Figure S10

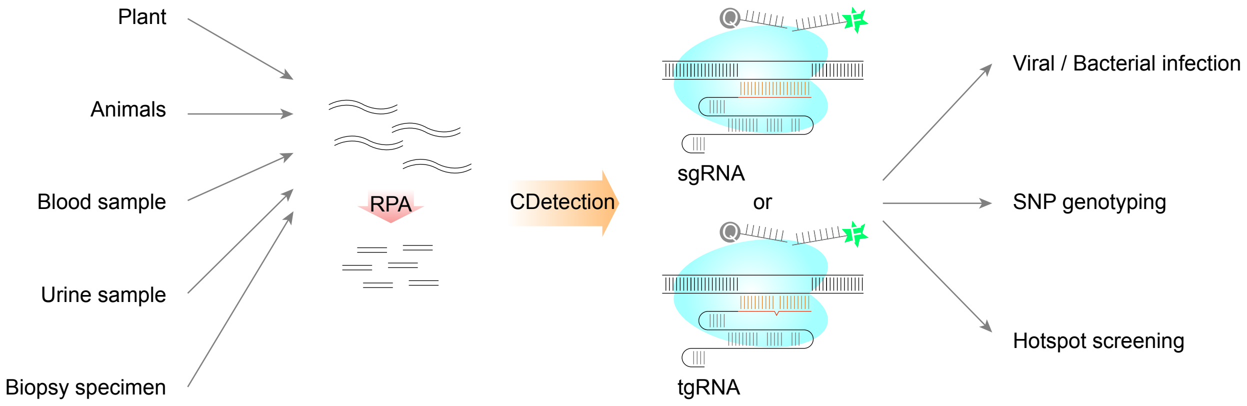
Plant
Animals
Blood sample
Urine sample
Biopsy specimen
RPA
CDetection
sgRNA
or
tgRNA
Q
F
Viral / Bacterial infection
SNP genotyping
Hotspot screening

Figure S10 Rapid and accurate diagnostic applications of CDetection. Schematics illustration of the wide applications of CDetection platform. Crude genomic DNAs are obtained from various samples via directly lysed for clinical diagnostics and quarantine inspection. For different purposes, DNAs with or without RPA were applied for Cas12b-based detection combined with sgRNA or tgRNA.

Figure S11

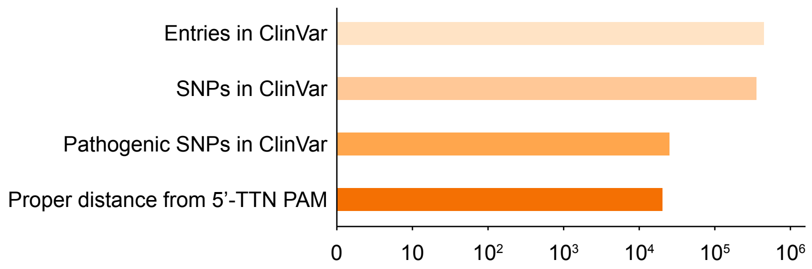

Entries in ClinVar
SNPs in ClinVar
Pathogenic SNPs in ClinVar
Proper distance from 5'-TTN PAM
0
10
10²
10³
10⁴
10⁵
10⁶

Figure S11 Genetic varaints from ClinVar that, in principle, can be detected by CDetection platform. The NCBI ClinVar database of human genetic variations and their corresponding phenotypes is searched for genetic disease that can be detected by CDetection platform. The results are filtered by imposing successive restrictions and the qualified number is shown on a logarithmic scale.
