# Supplementary material for: CDetection: CRISPR-Cas12b-based DNA detection with sub-attomolar sensitivity and single-base specificity
Source: Genome Biol. 2019 Jul 1;20:132. doi: 10.1186/s13059-019-1742-z (PMC6604390; doi:10.1186/s13059-019-1742-z)
Supplement: Supplementary file 2 — Table S1. Nucleic acids used in this study. Table S2. The components of buffers tested for Cas12b-mediated DNA detection in this study. (PDF 293 kb) [file 13059_2019_1742_MOESM2_ESM.pdf]

Table S1. Nucleic acids used in this study. The PAM sequences are colored in blue, mismatches in red and T7 promoter sequences in green, respectively. Protospacer and spacer sequences are underlined.

**DNA**	
Target 1[a]_NTS-50	TCCCAGAATGGCTTTCCCTCCCAGTCCCTTGGCTATGGAATATGTTAATG
Target 1[a]_TS-50	CATTAACATATTCCATAGCCAAGGGACTGGGAGGGAAAGCCATTCTGGGA
Target 1[a]_NTS-100	TCTGCTCAGTCCCAGAATGGCTTTCCCTCCCAGTCCCTTGGCTATGGAATATGTTAATGACTTTGATTTGATGAAGTTCGAAATAAAGCGGGAGCCTTCT
Target 1[a]_TS-100	AGAAGGCTCCCGCTTTATTTCGAACTTCATCAAATCAAAGTCATTAACATATTCCATAGCCAAGGGACTGGGAGGGAAAGCCATTCTGGGACTGAGCAGA
Target 2[a]_NTS-50	GCTGCTGTGAAGATTGTGGAGAGGAATGGCTTCAGTGATAAGATTAAAGT
Target 2[a]_TS-50	ACTTTAATCTTATCACTGAAGCCATTCCTCTCCACAATCTTCACAGCAGC
Target 2[a]_NTS-100	TAAGCCTATGGCTGAGGCTGCTGTGAAGATTGTGGAGAGGAATGGCTTCAGTGATAAGATTAAAGTCATTAACAAGCACTCCACTGAGGTGACAGTCGGA
Target 2[a]_TS-100	TCCGACTGTCACCTCAGTGGAGTGCTTGTTAATGACTTTAATCTTATCACTGAAGCCATTCCTCTCCACAATCTTCACAGCAGCCTCAGCCATAGGCTTA
Target 1[a]_NTS-100-mPAM	TCTGCTCAGTCCCAGAATGGCTAACCCTCCCAGTCCCTTGGCTATGGAATATGTTAATGACTTTGATTTGATGAAGTTCGAAATAAAGCGGGAGCCTTCT
Target 1[a]_TS-100-mPAM	AGAAGGCTCCCGCTTTATTTCGAACTTCATCAAATCAAAGTCATTAACATATTCCATAGCCAAGGGACTGGGAGGGTTAGCCATTCTGGGACTGAGCAGA
Target 1[a]_NTS-100-m1	TCTGCTCAGTCCCAGAATGGCTTTCGCTCCCAGTCCCTTGGCTATGGAATATGTTAATGACTTTGATTTGATGAAGTTCGAAATAAAGCGGGAGCCTTCT
Target 1[a]_TS-100-m1	AGAAGGCTCCCGCTTTATTTCGAACTTCATCAAATCAAAGTCATTAACATATTCCATAGCCAAGGGACTGGGAGCGAAAGCCATTCTGGGACTGAGCAGA
Target 1[a]_NTS-100-m3	TCTGCTCAGTCCCAGAATGGCTTTCCCACCCAGTCCCTTGGCTATGGAATATGTTAATGACTTTGATTTGATGAAGTTCGAAATAAAGCGGGAGCCTTCT

Target 1ª_TS-100-m3	AGAAGGCTCCCGCTTTATTTCGAACTTCATCAAATCAAAGTCATTAACATATTCCATAGCCAAGGGACTGGGTGGGAAAGCCATTCTGGGACTGAGCAGA
Target 1ª_NTS-100-m5	TCTGCTCAGTCCCAGAATGGCTTTCCCTCGCAGTCCCTTGGCTATGGAATATGTTAATGACTTTGATTTGATGAAGTTCGAAATAAAGCGGGAGCCTTCT
Target 1ª_TS-100-m5	AGAAGGCTCCCGCTTTATTTCGAACTTCATCAAATCAAAGTCATTAACATATTCCATAGCCAAGGGACTGCGAGGGAAAGCCATTCTGGGACTGAGCAGA
Target 1ª_NTS-100-m7	TCTGCTCAGTCCCAGAATGGCTTTCCCTCCCTGTCCCTTGGCTATGGAATATGTTAATGACTTTGATTTGATGAAGTTCGAAATAAAGCGGGAGCCTTCT
Target 1ª_TS-100-m7	AGAAGGCTCCCGCTTTATTTCGAACTTCATCAAATCAAAGTCATTAACATATTCCATAGCCAAGGGACAGGGAGGGAAAGCCATTCTGGGACTGAGCAGA
Target 1ª_NTS-100-m9	TCTGCTCAGTCCCAGAATGGCTTTCCCTCCCAGACCCTTGGCTATGGAATATGTTAATGACTTTGATTTGATGAAGTTCGAAATAAAGCGGGAGCCTTCT
Target 1ª_TS-100-m9	AGAAGGCTCCCGCTTTATTTCGAACTTCATCAAATCAAAGTCATTAACATATTCCATAGCCAAGGGTCTGGGAGGGAAAGCCATTCTGGGACTGAGCAGA
Target 1ª_NTS-100-m11	TCTGCTCAGTCCCAGAATGGCTTTCCCTCCCAGTCGCTTGGCTATGGAATATGTTAATGACTTTGATTTGATGAAGTTCGAAATAAAGCGGGAGCCTTCT
Target 1ª_TS-100-m11	AGAAGGCTCCCGCTTTATTTCGAACTTCATCAAATCAAAGTCATTAACATATTCCATAGCCAAGCGACTGGGAGGGAAAGCCATTCTGGGACTGAGCAGA
Target 1ª_NTS-100-m13	TCTGCTCAGTCCCAGAATGGCTTTCCCTCCCAGTCCCATGGCTATGGAATATGTTAATGACTTTGATTTGATGAAGTTCGAAATAAAGCGGGAGCCTTCT
Target 1ª_TS-100-m13	AGAAGGCTCCCGCTTTATTTCGAACTTCATCAAATCAAAGTCATTAACATATTCCATAGCCATGGGACTGGGAGGGAAAGCCATTCTGGGACTGAGCAGA
Target 1ª_NTS-100-m15	TCTGCTCAGTCCCAGAATGGCTTTCCCTCCCAGTCCCTTCGCTATGGAATATGTTAATGACTTTGATTTGATGAAGTTCGAAATAAAGCGGGAGCCTTCT
Target 1ª_TS-100-m15	AGAAGGCTCCCGCTTTATTTCGAACTTCATCAAATCAAAGTCATTAACATATTCCATAGCGAAGGGACTGGGAGGGAAAGCCATTCTGGGACTGAGCAGA
Target 1ª_NTS-100-m17	TCTGCTCAGTCCCAGAATGGCTTTCCCTCCCAGTCCCTTGGGTATGGAATATGTTAATGACTTTGATTTGATGAAGTTCGAAATAAAGCGGGAGCCTTCT

Target 1ª_TS-100-m17	AGAAGGCTCCCGCTTTATTTCGAACTTCATCAAATCAAAGTCATTAACATATTCCATACCCAAGGGACTGGGAGGGAAAGCCATTCTGGGACTGAGCAGA
Target 1ª_NTS-100-m19	TCTGCTCAGTCCCAGAATGGCTTTCCCTCCCAGTCCCTTGGCTTTGGAATATGTTAATGACTTTGATTTGATGAAGTTCGAAATAAAGCGGGAGCCTTCT
Target 1ª_TS-100-m19	AGAAGGCTCCCGCTTTATTTCGAACTTCATCAAATCAAAGTCATTAACATATTCCAAAGCCAAGGGACTGGGAGGGAAAGCCATTCTGGGACTGAGCAGA
Target 1ª_NTS-100-m1m2	TCTGCTCAGTCCCAGAATGGCTTTCGGTCCCAGTCCCTTGGCTATGGAATATGTTAATGACTTTGATTTGATGAAGTTCGAAATAAAGCGGGAGCCTTCT
Target 1ª_TS-100-m1m2	AGAAGGCTCCCGCTTTATTTCGAACTTCATCAAATCAAAGTCATTAACATATTCCATAGCCAAGGGACTGGGACCGAAAGCCATTCTGGGACTGAGCAGA
Target 1ª_NTS-100-m3m4	TCTGCTCAGTCCCAGAATGGCTTTCCCAGCCAGTCCCTTGGCTATGGAATATGTTAATGACTTTGATTTGATGAAGTTCGAAATAAAGCGGGAGCCTTCT
Target 1ª_TS-100-m3m4	AGAAGGCTCCCGCTTTATTTCGAACTTCATCAAATCAAAGTCATTAACATATTCCATAGCCAAGGGACTGGCTGGGAAAGCCATTCTGGGACTGAGCAGA
Target 1ª_NTS-100-m5m6	TCTGCTCAGTCCCAGAATGGCTTTCCCTCGGAGTCCCTTGGCTATGGAATATGTTAATGACTTTGATTTGATGAAGTTCGAAATAAAGCGGGAGCCTTCT
Target 1ª_TS-100-m5m6	AGAAGGCTCCCGCTTTATTTCGAACTTCATCAAATCAAAGTCATTAACATATTCCATAGCCAAGGGACTCCGAGGGAAAGCCATTCTGGGACTGAGCAGA
Target 1ª_NTS-100-m7m8	TCTGCTCAGTCCCAGAATGGCTTTCCCTCCCTCTCCCTTGGCTATGGAATATGTTAATGACTTTGATTTGATGAAGTTCGAAATAAAGCGGGAGCCTTCT
Target 1ª_TS-100-m7m8	AGAAGGCTCCCGCTTTATTTCGAACTTCATCAAATCAAAGTCATTAACATATTCCATAGCCAAGGGAGAGGGAGGGAAAGCCATTCTGGGACTGAGCAGA
Target 1ª_NTS-100-m9m10	TCTGCTCAGTCCCAGAATGGCTTTCCCTCCCAGAGCCTTGGCTATGGAATATGTTAATGACTTTGATTTGATGAAGTTCGAAATAAAGCGGGAGCCTTCT
Target 1ª_TS-100-m9m10	AGAAGGCTCCCGCTTTATTTCGAACTTCATCAAATCAAAGTCATTAACATATTCCATAGCCAAGGCTCTGGGAGGGAAAGCCATTCTGGGACTGAGCAGA
Target 1ª_NTS-100-m11m12	TCTGCTCAGTCCCAGAATGGCTTTCCCTCCCAGTCGGTTGGCTATGGAATATGTTAATGACTTTGATTTGATGAAGTTCGAAATAAAGCGGGAGCCTTCT

Target 1[a]_TS-100-m11m12	AGAAGGCTCCCGCTTTATTTCGAACTTCATCAAATCAAAGTCATTAACATATTCCATAGCCAACCGACTGGGAGGGAAAGCCATTCTGGGACTGAGCAGA
Target 1[a]_NTS-100-m13m14	TCTGCTCAGTCCCAGAATGGCTTTCCCTCCCAGTCCCAAGGCTATGGAATATGTTAATGACTTTGATTTGATGAAGTTCGAAATAAAGCGGGAGCCTTCT
Target 1[a]_TS-100-m13m14	AGAAGGCTCCCGCTTTATTTCGAACTTCATCAAATCAAAGTCATTAACATATTCCATAGCCTTGGGACTGGGAGGGAAAGCCATTCTGGGACTGAGCAGA
Target 1[a]_NTS-100-m15m16	TCTGCTCAGTCCCAGAATGGCTTTCCCTCCCAGTCCCTTCCCTATGGAATATGTTAATGACTTTGATTTGATGAAGTTCGAAATAAAGCGGGAGCCTTCT
Target 1[a]_TS-100-m15m16	AGAAGGCTCCCGCTTTATTTCGAACTTCATCAAATCAAAGTCATTAACATATTCCATAGGGAAGGGACTGGGAGGGAAAGCCATTCTGGGACTGAGCAGA
Target 1[a]_NTS-100-m17m18	TCTGCTCAGTCCCAGAATGGCTTTCCCTCCCAGTCCCTTGGGAATGGAATATGTTAATGACTTTGATTTGATGAAGTTCGAAATAAAGCGGGAGCCTTCT
Target 1_TS-100-m17m18	AGAAGGCTCCCGCTTTATTTCGAACTTCATCAAATCAAAGTCATTAACATATTCCATTCCCAAGGGACTGGGAGGGAAAGCCATTCTGGGACTGAGCAGA
Target 1_NTS-100-m19m20	TCTGCTCAGTCCCAGAATGGCTTTCCCTCCCAGTCCCTTGGCTTAGGAATATGTTAATGACTTTGATTTGATGAAGTTCGAAATAAAGCGGGAGCCTTCT
Target 1[a]_TS-100-m19m20	AGAAGGCTCCCGCTTTATTTCGAACTTCATCAAATCAAAGTCATTAACATATTCCTAAGCCAAGGGACTGGGAGGGAAAGCCATTCTGGGACTGAGCAGA
CaMV_NTS-100	TCAACTTCTTCTGCTTCTTGGATAAGTCGCAGATTTTAGATAGTTCTTCTTTTACTATCTTCGCCGCGAAACCTAAGCTGTAGATGCTGGTTCCGTTAGC
HPV16 site 1[b]_NTS-100	CGCCAGGGTTTTCCCAGTCACGACTTTGGTGCTATGGACTTTACTACATTACAGGCTAACAAAAGTGAAGTTCCACTCCTGTGTGAAATTGTTATCCGCT
HPV18 site 1[b]_NTS-100	CGCCAGGGTTTTCCCAGTCACGACTATGGTGCCATGGACTTTAGTACATTGCAAGATACTAAATGTGAGGTACCATTCCTGTGTGAAATTGTTATCCGCT
HPV16 site 2[b]_NTS-100	GTCGTAGGTACTCCTTAAAGTTAGTATTTTTATATGTAGTTTCTGAAGTAGATATGGCAGCACATAATGACATATTTGTACTGCGTGTAGTATCAACAAC
HPV18 site 2[b]_NTS-100	TACTGTGGTAGATACCACTCCCAGTACCAATTTAACAATATGTGCTTCTACACAGTCTCCTGTACCTGGGCAATATGATGCTACCAAATTTAAGCAGTAT

off-B101_NTS-100	CAGCGAGGTGGATTACCTGGTGTGCGTGGACGTGGACATGGAGTTCCGCGACCACGTGGGCGTGGAGATCCTGACTCCGCTGTTCGGCACCCTGCACCCC
on-B101_NTS-100	CAGCGAGGTGGATTACCTGGTGTGCGTGGACGTGGACATGGAGTTCCGCGACCATGTGGGCGTGGAGATCCTGACTCCGCTGTTCGGCACCCTGCACCCC
Off-O01_NTS-100	GCAAACTCACGTGGCGGCGCTTTGTGCTTGCAGGATGGTCTACCCCCAGCCAAAGGTGCTGACACCGTGGTGAGTAAAGTTACTGACACTGAAACTGAAC
ON-O01_NTS-100	GCAAACTCACGTGGCGGCGCTTTGCGCTTGCAGGATGGTCTACCCCCAGCCAAAGGTGCTGACACCGTGGTGAGTAAAGTTACTGACACTGAAACTGAAC
Off-O02/03_NTS-100	GTTCAGTTTCAGTGTCAGTAACTTTACTCACCACGGTGTCAGCACCTTTGGCTGGGGGTAGACCATCCTGCAAGCACAAAGCGCCGCCACGTGAGTTTGC
ON-O02/03_NTS-100	GTTCAGTTTCAGTGTCAGTAACTTTACTCACCACGGTGTCAGCACCTTTGACTGGGGGTAGACCATCCTGCAAGCACAAAGCGCCGCCACGTGAGTTTGC
BRCA1_3232WT_NTS-100	GTACAGTGAGCACAATTAGCCGTAATAACATTAGAGAAAATGTTTTTAAAGAAGCCAGCTCAAGCAATATTAATGAAGTAGGTTCCAGTACTAATGAAGT
BRCA1_3232A>G_NTS-100	GTACAGTGAGCACAATTAGCCGTAATAACATTAGAGAAAATGTTTTTAAAGGAGCCAGCTCAAGCAATATTAATGAAGTAGGTTCCAGTACTAATGAAGT
BRCA1_3537WT_NTS-100	CAGATTTCTCTCCATATCTGATTTCAGATAACTTAGAACAGCCTATGGGAAGTAGTCATGCATCTCAGGTTTGTTCTGAGACACCTGATGACCTGTTAGA
BRCA1_3537A>G_NTS-100	CAGATTTCTCTCCATATCTGATTTCAGATAACTTAGAACAGCCTATGGGAAGTGGTCATGCATCTCAGGTTTGTTCTGAGACACCTGATGACCTGTTAGA
TP53_856WT_NTS	CTTTGAGGTGCGTGTTTGTGCCTGTCCTGGGAGAGACCGGCGCACAGAGGAAGAGAATCTCCGCAAGAAAGGGGAGCCTCACCACGAGCTGCCCCCAGG
TP53_856G>A_NTS	CTTTGAGGTGCGTGTTTGTGCCTGTCCTGGGAGAGACCGGCGCACAGAGAAAGAGAATCTCCGCAAGAAAGGGGAGCCTCACCACGAGCTGCCCCCAGG
dsActivator 1[b]_NTS	CGCCAGGGTTTTCCCAGTCACGACTTTAGACCAGGATGGGCACCACCCTCCTGTGTGAAATTGTTATCCGCT
dsActivator 2[b]_NTS	CGCCAGGGTTTTCCCAGTCACGACTTTAGATCGTTACGCTAACTATGATCCTGTGTGAAATTGTTATCCGCT
dsActivator 3[c]_NTS-100	CGCCAGGGTTTTCCCAGTCACGACGTACCCGGGGATCCTTTAGAGAAGTCATTTAATAAGGCCACTGTTAAAAAGCTCCTGTGTGAAATTGTTATCCGCT

DNMT1 site 1[d]_NTS-100	CGCCAGGGTTTTCCCAGTCACGACCCCTGGGGCCGTTTCCCTCACTCCTGCTCGGTGAATTTGGCTCAGCAGGCACTCCTGTGTGAAATTGTTATCCGCT
DNMT1 site 2[d]_NTS-100	CGCCAGGGTTTTCCCAGTCACGACTCTGGGGACCGTTTGAGGAGTGTTCAGTCTCCGTGAACGTTCCCTTAGCACTTCCTGTGTGAAATTGTTATCCGCT
DNMT1 site 3[d]_NTS-100	CGCCAGGGTTTTCCCAGTCACGACATCAGTACGTTAATGTTTCCTGATGGTCCATGTCTGTTACTCGCCTGTCAAGTCCTGTGTGAAATTGTTATCCGCT
DNMT1 site 4[d]_NTS-100	CGCCAGGGTTTTCCCAGTCACGACTGACTTTTCCTTTTATTTCCCTTCAGCTAAAATAAAGGAGGAGGAAGCTGCTTCCTGTGTGAAATTGTTATCCGCT
EMX2 site 1[d]_NTS-100	CGCCAGGGTTTTCCCAGTCACGACGCCGTTTGTACTTTGTCCTCCGGTTCTGGAACCACACCTTCACCTGGGCCAGTCCTGTGTGAAATTGTTATCCGCT
HERC2>A[c]_NTS-100	CGCCAGGGTTTTCCCAGTCACGACAGAGGCGAGGCCAGTTTCATTTGAGCATTAAATGTCAAGTTCTGCACGCTATTCCTGTGTGAAATTGTTATCCGCT
HERC2>G[c]_NTS-100	CGCCAGGGTTTTCCCAGTCACGACAGAGGCGAGGCCAGTTTCATTTGAGCATTAAGTGTCAAGTTCTGCACGCTATTCCTGTGTGAAATTGTTATCCGCT
BRCA1_3232-sgPOT-1	CTGCTCCCCTCATGGCCCCACTTGCTCCTTCTGGACCACTTGAAGGAACCAGCTCATGCTATTTTTCATTCCCACCTTTATGTCATCTTAGAAACATAGT
BRCA1_3232-sgPOT-2	ATCATGGGAACTGCAGGAAGAGGTTCCCAGTAGAGGATCTTCAAGGAGCCTGCACAAGCAACCAATGAAACAAAGAGCTAGTAATTGTGCATCTGACATT
BRCA1_3232-sgPOT-3	GATTTTTATGGAGGTTGACTTTGTGTGTGCATTGGAGTTTTGAAGGTGCCATCTCAGGCAATTCACCCCCGAGGAGGGAAACATGGTTCTCAGTGCAGCT
BRCA1_3232-sgPOT-4	GTCTGTCTATAGGGGTTGGGGGAGAAAGAAAGAATTTATTTTAAGGAATCAGCTCATGCAATAGTGGGGACTTGCAAGTCCGAAATCCATAGGCTGGTAT
BRCA1_3232-sgPOT-5	AAGATAACAATGCCCATGAGTGTGGCAGGGTTCAGACTGTTTAAGGAGCAAGCTCAAAGAATCTTCCTCACTGGGTGGGGGCCGAGGAGAATGGCAACCA
BRCA1_3232-sgPOT-6/tgPOT-3	TATTCACTTCATGTAGGTGCTGAATTTAATTGAGTTAGATTCTAGGAGAAAGCTCAAGCAATGCCTAACCAAGGAAGCCGTGGATTCCCATTCAGAGCCA
BRCA1_3232-sgPOT-7	AAGAATTTCAGCTGAATAACAACAAAAAAGTATTAGAAATTGAAGGAGTCAGCTAAAGCAGTACTTAGAGAAAAATTTATAGCTTTAAGTATTTATACTA

BRCA1_3232-sgPOT-8	TTACAGATGCTTCCTCAGCAACGGAAAGCCATAGCTAAGTTCAAGGAGCCAGCCCACGCATTAGCATTTCAGCAGAAATTCCACAGGTGACCAGTGTGTT
BRCA1_3232-sgPOT-9/tgPOT-4	CTCACAAAGATTACAAACCACAAAAGGAAATAAAGCTTTTTGAAGGAGTCAGCTAAAGCAATGGAGGAATGTATACCCCAAAACTGAACATAGCAATCTC
BRCA1_3232-sgPOT-10/tgPOT-5	ATTTTGAACTGAATGAAAATGAAAACGCCAGATCAAAATTTGTAGGAGACAGCTAAAGCAATGCTTGAGGAAATGTTCTGGCATTAAATGTTTTTTATAA
BRCA1_3232-sgPOT-11	ACCCTGACTGATACAGATAACCTAGCCCAAGAGTCTGGATTCAATGTGCCAGCTCAAGCAGTTCTTAATGATTCTAACAGTTGCTCCACTATTAAACTAT
BRCA1_3232-sgPOT-12	GCACCATATGGAAGTTATCAAGGCTTATAGCTTGCTTTCTTCAAGGAGGCAGCTCAAGCTGTACCTGGACCCCCTTGAACCACAGCTGAACAGCTGGGAT
BRCA1_3232-sgPOT-13/tgPOT-6	GCAGCCGGGTTCTGCCCTCGTTCGGGGAACTCTGCCAGGTTTCAGGAGCCAGCTCAGGCACTTCGCCCCCTCCTCAGAGGCTGCCTCTTGAGCCTTCAGT
BRCA1_3232-sgPOT-14	CATATCATTGCTGAGTATTCCTTTGGAGATTTCCTTCAATTGAAGAAGCCACCTCATGCAATGTCATGTCTTATTCTCAAGAGCAGCTCATGTCCAGTGA
BRCA1_3232-sgPOT-15	TTCTAGTTGTTTTCTCTTTTGGAAATACATTAATGCTTCTTCAAGGAGCCAGGCAAAGCAATCAGATCACTCACTGTAATCCTTATGAGGAAGTGTATAT
BRCA1_3232-sgPOT-16	AATATGCTGGAGATTTATGGGGACTATCTATGATTGTGGTTCAGGGAGCCTGCTCAAGCAAACATAAATCTTGGTAAAAGAGTCAGTCAAGCCATATAAA
BRCA1_3232-sgPOT-17	ATGTCACAAACGTTGTGCAGTCCCTATGAGTTTCCCACATTGAATGAGCCAGCTTAAACAATATAAGCCTAAACTTCAAAGCTTGATAAATACCCTTCGG
BRCA1_3232-sgPOT-18	ACAATTGTTATAGGGAAGGCAAAATTCCACCTCCATTCATTTAGGGATCCAGCTCAAGAAATAAATTTACATAAGATAGATTAAAATGAATAAGAGCATA
BRCA1_3232-sgPOT-19	GACCTAGCTCCTAGGATGCTTGAGGCTGAAGGAATGGTGTTTAAGGAGAAATCTCAAGCAATGCCAAGTTCCAGTCAGGTAGAATTAGAGTAGCTGAAAC
BRCA1_3232-sgPOT-20	CTTAGGAAGCTCCAAGTTATAGAACCTTCTCAAGGGGTGTTAAAGGAGCCATCTGAAACAATAGGATGCTTCCTTAAGACAGGAGCCAAGATCTTGGTAT
BRCA1_3232-tgPOT-1	GAAAGGTAAATGCTAGGAACAGGTGGCATCACCAGATACTTTTAGGAGCCAGCTCACGTGATGTCTTGGCCTTTATGTTATAGGTAGGTAAACAAACTAT

BRCA1_3232-tgPOT-2	CTTTTAGATAAATGGAAATGAAGACAAACATACCAAATCTTATAGGATGCAGCTTAAGCAATGTCTATGAGAATTTTTTTTTAATTTTATTATTATTATA
BRCA1_3232-tgPOT-7	TTTGGAGCAAGTACCAAATGCCAAGCACTGTATTATGCATTCTAGAAGCCATCTCATGCAATCCTCACAAAAACTCAAAAAGGTGGTGTTTTACAGATGA
BRCA1_3232-tgPOT-8	ATGATTGCCCGCCAAGATTTTCCTTTTTTTTTTTTTTTTTTTTTGGTACCAGCTCAAGCAATTCTCCTGCCTCAGCCTCCCAAGTAGCTGAGATTACAGG
BRCA1_3232-tgPOT-9	TGCGTTGGGCAAACACAAAGATGCTATTTGTAAAGCAAATTTTCAGAGCCAGCTCAAGAAATGGAAAATACTCACCTAACTCGTGAGGTGCCTGTGATAA
BRCA1_3232-tgPOT-10	AAAGCCTGGGAGTGATTAACAACCACTGCATAGCAAAACTTTTAGGAGTCAGCTAAAGCACTATGGACAGGGAAATTGTGGACTTAAAAGCTCCTGTAAG
BRCA1_3232-tgPOT-11	GAAAATTTCTGAAATGAATTAAAATGAGAATATCAAAATTTGTAGGATACAGCTAAAGCAATATTTCAGAGAGAAATTCATAGTATTATATTAGTATTCA
BRCA1_3232-tgPOT-12	GCTGCAGAGCCGGTGGTTTTCCTATCACTACAGGTGGGGTTTTAGGAGCCACCTCCAGCAAGAAGCCACAAACCGGGCGTCCTTACCTGACGCGGATGCA
BRCA1_3232-tgPOT-13	CCTTTCAAAGAGTGCATTTGCTATCACAAATGGTGCTTCTTTTAGGACACAGCCCAAGCAATATTGCCTGCTTATTACCCCAAATGAAACATGAAGAATC
BRCA1_3232-tgPOT-14	AATATTTTGAACTGAATGAAAATGAAAAAACTTCGAAATTTGTGGGATGCAGCTCAAGCAATGCTTACAGGAAACCGACAGCATTAAATGCTTATATTAG
BRCA1_3232-tgPOT-15	AAATTATTTTAACCTGAATGATAATGAAAACATCAAAATTTGTAGGATGCAGCTAAAGCAATGTTAGAGGAAATATATAGCCTTATATGTATATTTTAGA
BRCA1_3232-tgPOT-16	TTTTGAATTAAATAAAAATGATACACAGCTTATCAAAAGTTGTAGGATGCAGCTAAAGCAATACTTAGAGGGAATTTATAGTTTGAAATGCTTATTTAGA
BRCA1_3232-tgPOT-17	TTTTGTTTGAGTGATAATAAATATATTGTATGTCACAACTTGTGGGAGGCAGCTCAAACAATACTTAGAAGAAAATATATAGTGTTAAATGCATATATTC
BRCA1_3232-tgPOT-18	TCTCTCTCTTTCTTTCCAAGAGGCCAATTTCTACCTATATTTTAGGATCCAGCTCAAGCATCACCTCCTCCACAAAGCTTTTTCTAACCCCCTCATCAGC
BRCA1_3232-tgPOT-19	GGTTAGCTGGATTTGTCCGTATTGCAGATGAGAGGGAGTTTTTAGGACCCAGTTCCAGCAATTTTTCCTCCTCTGCACAGGTGCCATGCATCACCTTTTC

BRCA1_3232-tgPOT-20	TAATGCATAACTTCATTTAAAAAGTTTTGAAAATATAGTTTCTTGGAGCTAGCTCAAGCATTAATTGCACAGATTGTTAAAATAGTCTGTATGCTATGGA
BRCA1_3232-tgPOT-21	CATTTTGTTCTGAATAAAAAGAAGACTACATATCAAAATTTGTAGGATGCAGCTAAAGCAATGCTTAGAGGGGAATTTATAGTTTTAAACACATGTTAAA
BRCA1_3232-tgPOT-22	TCGAACATACCCAAGAGTAGAGAGAACCATACAATAAACTTTTGTGAGCCAGCTCCAGCAATTATCCATGTTGCCATCCTGTTTCATGTATCCCTCCCCT
BRCA1_3232-tgPOT-23	TATATTGAACTGAAAGAAAAGGAAAATACAACATAACATTTATAGGATACAGCTAAAGCAATGTGGCAAGAGAAATTTACAGCACCAAATGCTTAGATCA
TP53_856-sgPOT-1/tgPOT-1	AAAGTTATTTGGAATATTTCTGCTCGACAGGTCCAATATTTTTCTTCCTCTGTGTGCCGGTCATAAGGAAATCTTCAGGACGCCAGTGATGCCAGCTAAA
TP53_856-sgPOT-2	GAGGAGTTTGACTTTAGCATTAGCACATGAGCAAGCGTATTCTCTTTCTCTGTGCATCTGTCTAATAAAACAATAGGAATATAGCACAAGTAAACTCTAT
TP53_856-sgPOT-3	TGGCTCCCCTCTGTCCATCTGCAAGACAGACAGCTCTGGTTCTCTTTCTCTGGGCGCCAGTGCACCCCCAAGCCATGTCAAGCTGAGCCCAGGAGTGCCT
TP53_856-sgPOT-4/tgPOT-4	GCACCTCCACGGCACTCCCAACTTTTTGTCCCCCAGACGTTTGCCTTCTCTGTGCGCTGGTCAGACGGCAGCGACACCTTCGTGCGCAGGAGTTGGGACG
TP53_856-sgPOT-5	TGGAACTTGCCAACAGATTTTTGCTTTTTGTATCTGCCCTTCTCTTTCTCTGTCTGCTGGTCTTTCCCTGCCTCTGCCAGCTGCCCATGCTGCTATTTTC
TP53_856-sgPOT-6	CAGCCAAAAACAGCAGTTCATTTGGGCATGTTTTCAATTTTCTATTTCTCTGGGCACCGGTCCCCATGGCCAGGTCAAGCTGTTCAACGCTCACACCAGC
TP53_856-sgPOT-7	GTTTATTTCTGGGTTTTATATTTGATTCTACTGATCTACTTGTCTTTCTCTGTGCCACGGCCTCACTATTCTAATTTTAATTGCTTTATTATAAGTCTTA
TP53_856-sgPOT-8	ATCTTAGGACTCACAGTCTCCGGGCCTTTCTCTTCATCATTCTCTTTCTCTGTGCTCCCCTCTGTCTGGGTTTCAATCTCAGAACGATTACTTGTTTTCT
TP53_856-sgPOT-9	GAACTCCACATGAGATCACAGGTGCAGGATTAGGGAAGCTTCTCTTTTTCTGGGCCCCGGTCCATGCTAGCGGGGTTTCAGTCACTTGTCAATGGGTCCA
TP53_856-sgPOT-10	GAACTCCACATGAGACCACAGGTGCAGGATTAGGGAAGCTTCTCTTTTTCTGGGCCCCGGTCCATGCTAGCAGGGTTTCAGTCACTTGTCAATGGGTCCA

TP53_856-tgPOT-2	CTGTAGAGAACTAATGTAGTCTACACTGTACACTTGATGTTCAGTTTCTCTGTGCAGCGGTCACTTGTGAATTTTCAGGAGCCTTGCACAGTGAAGTAAC
TP53_856-tgPOT-3	CAACCCAACAGATTAGTTTGAAAACAATGTACGCCTATGTTTACTTTCTCTGTCCGCCTCTCCCTCAGGACCAGGTGCCTAGCTGACTTAGCACATTCTC
T7-AasgRNA-M13mp18	TAATACGACTCACTATAGGGTCTAAAGGACAGAATTTTTCAACGGGTGTGCCAATGGCCACTTTCCAGGTGGCAAAGCCCGTTGAACTTCAAGCGAAGTGGCACGATCGTTACGCTAACTATGA
T7-AasgRNA-Target 1	TAATACGACTCACTATAGGGTCTAAAGGACAGAATTTTTCAACGGGTGTGCCAATGGCCACTTTCCAGGTGGCAAAGCCCGTTGAACTTCAAGCGAAGTGGCACCCTCCCAGTCCCTTGGCTAT
T7-AasgRNA-Target 2	TAATACGACTCACTATAGGGTCTAAAGGACAGAATTTTTCAACGGGTGTGCCAATGGCCACTTTCCAGGTGGCAAAGCCCGTTGAACTTCAAGCGAAGTGGCACTGGAGAGGAATGGCTTCAGT
T7-AasgRNA-HPV16_site_1	TAATACGACTCACTATAGGGTCTAAAGGACAGAATTTTTCAACGGGTGTGCCAATGGCCACTTTCCAGGTGGCAAAGCCCGTTGAACTTCAAGCGAAGTGGCACCTACATTACAGGCTAACAAA
T7-AasgRNA-HPV18_site_1	TAATACGACTCACTATAGGGTCTAAAGGACAGAATTTTTCAACGGGTGTGCCAATGGCCACTTTCCAGGTGGCAAAGCCCGTTGAACTTCAAGCGAAGTGGCACGTACATTGCAAGATACTAAA
T7-AasgRNA-HPV16_site_2	TAATACGACTCACTATAGGGTCTAAAGGACAGAATTTTTCAACGGGTGTGCCAATGGCCACTTTCCAGGTGGCAAAGCCCGTTGAACTTCAAGCGAAGTGGCACTGAAGTAGATATGGCAGCAC
T7-AasgRNA-HPV18_site_2	TAATACGACTCACTATAGGGTCTAAAGGACAGAATTTTTCAACGGGTGTGCCAATGGCCACTTTCCAGGTGGCAAAGCCCGTTGAACTTCAAGCGAAGTGGCACACAATATGTGCTTCTACACA
T7-AasgRNA-CaMV	TAATACGACTCACTATAGGGTCTAAAGGACAGAATTTTTCAACGGGTGTGCCAATGGCCACTTTCCAGGTGGCAAAGCCCGTTGAACTTCAAGCGAAGTGGCACCTATCTTCGCCGCGAAACCT
T7-AasgRNA-B101	TAATACGACTCACTATAGGGTCTAAAGGACAGAATTTTTCAACGGGTGTGCCAATGGCCACTTTCCAGGTGGCAAAGCCCGTTGAACTTCAAGCGAAGTGGCACCGCGACCATGTGGGCGTGGA
T7-AatgRNA-B101	TAATACGACTCACTATAGGGTCTAAAGGACAGAATTTTTCAACGGGTGTGCCAATGGCCACTTTCCAGGTGGCAAAGCCCGTTGAACTTCAAGCGAAGTGGCACCGCGACCATATGGGCGTGGA
T7-AasgRNA-O01	TAATACGACTCACTATAGGGTCTAAAGGACAGAATTTTTCAACGGGTGTGCCAATGGCCACTTTCCAGGTGGCAAAGCCCGTTGAACTTCAAGCGAAGTGGCACCGCTTGCAGGATGGTCTACC
T7-AatgRNA-O01	TAATACGACTCACTATAGGGTCTAAAGGACAGAATTTTTCAACGGGTGTGCCAATGGCCACTTTCCAGGTGGCAAAGCCCGTTGAACTTCAAGCGAAGTGGCACCACTTGCAGGATGGTCTACC

T7-AasgRNA-O02/03	TAATACGACTCACTATAGGGTCTAAAGGACAGAATTTTTCAACGGGTGTGCCAATGGCCACTTTCCAGGTGGCAAAGCCCGTTGAACTTCAAGCGAAGTGGCACACTGGGGGTAGACCATCCTG
T7-AatgRNA-O02/03	TAATACGACTCACTATAGGGTCTAAAGGACAGAATTTTTCAACGGGTGTGCCAATGGCCACTTTCCAGGTGGCAAAGCCCGTTGAACTTCAAGCGAAGTGGCACAATGGGGGTAGACCATCCTG
T7-AasgRNA-BRCA1_3232	TAATACGACTCACTATAGGGTCTAAAGGACAGAATTTTTCAACGGGTGTGCCAATGGCCACTTTCCAGGTGGCAAAGCCCGTTGAACTTCAAGCGAAGTGGCACAAGGAGCCAGCTCAAGCAAT
T7-AatgRNA-BRCA1_3232-1	TAATACGACTCACTATAGGGTCTAAAGGACAGAATTTTTCAACGGGTGTGCCAATGGCCACTTTCCAGGTGGCAAAGCCCGTTGAACTTCAAGCGAAGTGGCACTAGGAGCCAGCTCAAGCAAT
T7-AatgRNA-BRCA1_3232-2	TAATACGACTCACTATAGGGTCTAAAGGACAGAATTTTTCAACGGGTGTGCCAATGGCCACTTTCCAGGTGGCAAAGCCCGTTGAACTTCAAGCGAAGTGGCACAAGGACCCAGCTCAAGCAAT
T7-AatgRNA-BRCA1_3232-3	TAATACGACTCACTATAGGGTCTAAAGGACAGAATTTTTCAACGGGTGTGCCAATGGCCACTTTCCAGGTGGCAAAGCCCGTTGAACTTCAAGCGAAGTGGCACAAGGAGCCAGGTCAAGCAAT
T7-AatgRNA-BRCA1_3232-4	TAATACGACTCACTATAGGGTCTAAAGGACAGAATTTTTCAACGGGTGTGCCAATGGCCACTTTCCAGGTGGCAAAGCCCGTTGAACTTCAAGCGAAGTGGCACAAGGAGCCAGCTCAACCAAT
T7-AasgRNA-BRCA1_3537	TAATACGACTCACTATAGGGTCTAAAGGACAGAATTTTTCAACGGGTGTGCCAATGGCCACTTTCCAGGTGGCAAAGCCCGTTGAACTTCAAGCGAAGTGGCACGAACAGCCTATGGGAAGTGG
T7-AatgRNA-BRCA1_3537-1	TAATACGACTCACTATAGGGTCTAAAGGACAGAATTTTTCAACGGGTGTGCCAATGGCCACTTTCCAGGTGGCAAAGCCCGTTGAACTTCAAGCGAAGTGGCACGAACAGCCTATGGGAAGTGG
T7-AatgRNA-BRCA1_3537-2	TAATACGACTCACTATAGGGTCTAAAGGACAGAATTTTTCAACGGGTGTGCCAATGGCCACTTTCCAGGTGGCAAAGCCCGTTGAACTTCAAGCGAAGTGGCACCAACAGCCTATGGGAAGTGG
T7-AatgRNA-BRCA1_3537-3	TAATACGACTCACTATAGGGTCTAAAGGACAGAATTTTTCAACGGGTGTGCCAATGGCCACTTTCCAGGTGGCAAAGCCCGTTGAACTTCAAGCGAAGTGGCACGAACACCCTATGGGAAGTGG
T7-AatgRNA-BRCA1_3537-4	TAATACGACTCACTATAGGGTCTAAAGGACAGAATTTTTCAACGGGTGTGCCAATGGCCACTTTCCAGGTGGCAAAGCCCGTTGAACTTCAAGCGAAGTGGCACGAACAGCCTAAGGGAAGTGG
T7-AasgRNA-TP53_856	TAATACGACTCACTATAGGGTCTAAAGGACAGAATTTTTCAACGGGTGTGCCAATGGCCACTTTCCAGGTGGCAAAGCCCGTTGAACTTCAAGCGAAGTGGCACTCTTTCTCTGTGCGCCGGTC
T7-AatgRNA-TP53_856-1	TAATACGACTCACTATAGGGTCTAAAGGACAGAATTTTTCAACGGGTGTGCCAATGGCCACTTTCCAGGTGGCAAAGCCCGTTGAACTTCAAGCGAAGTGGCACACTTTCTCTGTGCGCCGGTC

T7-AatgRNA-TP53_856-2	TAATACGACTCACTATAGGGTCTAAAGGACAGAATTTTTCAACGGGTGTGCCAATGGCCACTTTCCAGGTGGCAAAGCCCGTTGAACTTCAAGCGAAGTGGCACTCTTTCTGTGTGCGCCGGTC
T7-AatgRNA-TP53_856-3	TAATACGACTCACTATAGGGTCTAAAGGACAGAATTTTTCAACGGGTGTGCCAATGGCCACTTTCCAGGTGGCAAAGCCCGTTGAACTTCAAGCGAAGTGGCACTCTTTCTCTGAGCGCCGGTC
T7-AasgRNA-dsActivator-1	TAATACGACTCACTATAGGGTCTAAAGGACAGAATTTTTCAACGGGTGTGCCAATGGCCACTTTCCAGGTGGCAAAGCCCGTTGAACTTCAAGCGAAGTGGCACGACCAGGATGGGCACCACCC
T7-AasgRNA-dsActivator-2	TAATACGACTCACTATAGGGTCTAAAGGACAGAATTTTTCAACGGGTGTGCCAATGGCCACTTTCCAGGTGGCAAAGCCCGTTGAACTTCAAGCGAAGTGGCACGATCGTTACGCTAACTATGA
T7-AasgRNA-dsActivator-3	TAATACGACTCACTATAGGGTCTAAAGGACAGAATTTTTCAACGGGTGTGCCAATGGCCACTTTCCAGGTGGCAAAGCCCGTTGAACTTCAAGCGAAGTGGCACGAGAAGTCATTTAATAAGGC
T7-AasgRNA-DNMT1-1	TAATACGACTCACTATAGGGTCTAAAGGACAGAATTTTTCAACGGGTGTGCCAATGGCCACTTTCCAGGTGGCAAAGCCCGTTGAACTTCAAGCGAAGTGGCACCCTCACTCCTGCTCGGTGAA
T7-AasgRNA-DNMT1-2	TAATACGACTCACTATAGGGTCTAAAGGACAGAATTTTTCAACGGGTGTGCCAATGGCCACTTTCCAGGTGGCAAAGCCCGTTGAACTTCAAGCGAAGTGGCACAGGAGTGTTCAGTCTCCGTG
T7-AasgRNA-DNMT1-3	TAATACGACTCACTATAGGGTCTAAAGGACAGAATTTTTCAACGGGTGTGCCAATGGCCACTTTCCAGGTGGCAAAGCCCGTTGAACTTCAAGCGAAGTGGCACCTGATGGTCCATGTCTGTTA
T7-AasgRNA-DNMT1-4	TAATACGACTCACTATAGGGTCTAAAGGACAGAATTTTTCAACGGGTGTGCCAATGGCCACTTTCCAGGTGGCAAAGCCCGTTGAACTTCAAGCGAAGTGGCACTTTCCCTTCAGCTAAAATAA
T7-AasgRNA-EMX1-2	TAATACGACTCACTATAGGGTCTAAAGGACAGAATTTTTCAACGGGTGTGCCAATGGCCACTTTCCAGGTGGCAAAGCCCGTTGAACTTCAAGCGAAGTGGCACTCCTCCGGTTCTGGAACCAC
T7-AasgRNA-HERC2A	TAATACGACTCACTATAGGGTCTAAAGGACAGAATTTTTCAACGGGTGTGCCAATGGCCACTTTCCAGGTGGCAAAGCCCGTTGAACTTCAAGCGAAGTGGCACAGCATTAAATGTCAAGTTCT
T7-AasgRNA-HERC2G	TAATACGACTCACTATAGGGTCTAAAGGACAGAATTTTTCAACGGGTGTGCCAATGGCCACTTTCCAGGTGGCAAAGCCCGTTGAACTTCAAGCGAAGTGGCACAGCATTAAGTGTCAAGTTCT
T7-crRNA-M13mp18-F	TAATACGACTCACTATAGGATTTCTACTATTGTAGATGATCGTTACGCTAACTATGAGGG
T7-crRNA-M13mp18-R	CCCTCATAGTTAGCGTAACGATCATCTACAATAGTAGAAATCCTATAGTGAGTCGTATTA
T7-crRNA-HPV16-2F	TAATACGACTCACTATAGGATTTCTACTAAGTGTAGATTGAAGTAGATATGGCAGCACGGG
T7-crRNA-HPV16-2R	CCCGTGCTGCCATATCTACTTCAATCTACACTTAGTAGAAATCCTATAGTGAGTCGTATTA

T7-crRNA-HPV18-2F	TAATACGACTCACTATAGGATTTCTACTAAGTGTAGATACAATATGTGCTTCTACACAGGG
T7-crRNA-HPV18-2R	CCCTGTGTAGAAGCACATATTGTATCTACACTTAGTAGAAATCCTATAGTGAGTCGTATTA
T7-sgRNA-M13mp18	TAATACGACTCACTATAGGGTCTAAAGGACAGAATTTTTCAACGGGTGTGCCAATGGCCACTTTCCAGGTGGCAAAGCCCGTTGAACTTCAAGCGAAGTGGCACGATCGTTACGCTAACTATGA
T7-promoter-F	TAATACGACTCACTATAGG
T7-crRNA-BRCA1_3232-R	ATTGCTTGAGCTGGCTCCTTATCTACAATAGTAGAAATCCTATAGTGAGTCGTATTA
T7-crRNA-TP53_856-R	GACCGGCGCACAGAGAAAGAATCTACAATAGTAGAAATCCTATAGTGAGTCGTATTA
T7-crRNA-HPV16-1R	TTTGTTAGCCTGTAATGTAGATCTACAATAGTAGAAATCCTATAGTGAGTCGTATTA
T7-crRNA-HPV18-1R	TTTAGTATCTTGCAATGTACATCTACAATAGTAGAAATCCTATAGTGAGTCGTATTA
T7-crRNA-dsActivator-1R	GGGTGGTGCCCATCCTGGTCATCTACAATAGTAGAAATCCTATAGTGAGTCGTATTA
T7-crRNA-dsActivator-2R	TCATAGTTAGCGTAACGATCATCTACAATAGTAGAAATCCTATAGTGAGTCGTATTA
T7-crRNA-dsActivator-3R	GCCTTATTAAATGACTTCTCATCTACAATAGTAGAAATCCTATAGTGAGTCGTATTA
T7-crRNA-DNMT1-1R	TTCACCGAGCAGGAGTGAGGATCTACAATAGTAGAAATCCTATAGTGAGTCGTATTA
T7-crRNA-DNMT1-2R	CACGGAGACTGAACACTCCTATCTACAATAGTAGAAATCCTATAGTGAGTCGTATTA
T7-crRNA-DNMT1-3R	TAACAGACATGGACCATCAGATCTACAATAGTAGAAATCCTATAGTGAGTCGTATTA
T7-crRNA-DNMT1-4R	TTATTTTAGCTGAAGGGAAAATCTACAATAGTAGAAATCCTATAGTGAGTCGTATTA
T7-crRNA-EMX1-2R	GTGGTTCCAGAACCGGAGGAATCTACAATAGTAGAAATCCTATAGTGAGTCGTATTA
T7-crRNA-HERC2G-R	AGCATTAAATGTCAAGTTCTATCTACAATAGTAGAAATCCTATAGTGAGTCGTATTA
T7-crRNA-HERC2A-R	AGAACTTGACACTTAATGCTATCTACAATAGTAGAAATCCTATAGTGAGTCGTATTA
**Primers**	
Target 1-F	TCTGCTCAGTCCCAGAATGGC
Target 1-R	AGAAGGCTCCCGCTTTATTTCG
Target 2-F	TAAGCCTATGGCTGAGGCTGC
Target 2-R	TCCGACTGTCACCTCAGTGGAG
CaMV-F	GCTAACGGAACCAGCATCTACA
CaMV-R	TCAACTTCTTCTGCTTCTTGG

T7-AasgRNA-HPV16-R	GTGCTGCCATATCTACTTCAGTGCCACTTCGCTTGAAGTTCA
T7-AasgRNA-HPV18-R	TGTGTAGAAGCACATATTGTGTGCCACTTCGCTTGAAGTTCA
HPV16_site_2-F	GTTGTTGATACTACACGCAGTA
HPV16_site_2-R	GTCGTAGGTACTCCTTAAAGTT
HPV18_site_2-F	TACTGTGGTAGATACCACTCCC
HPV18_site_2-R	ATACTGCTTAAATTTGGTAGCA
B101-F	CAGCGAGGTGGATTACCTGGT
B101-R	GGGGTGCAGGGTGCCGAA
O01/02/03-F	GCAAACTCACGTGGCGGC
O01/02/03-R	GTTCAGTTTCAGTGTCAGTAAC
BRCA1_3232-F	GTACAGTGAGCACAATTAGCCG
BRCA1_3232-R	ACTTCATTAGTACTGGAACCTACTTC
BRCA1_3537-F	CAGATTTCTCTCCATATCTGATTTCAG
BRCA1_3537-R	TCTAACAGGTCATCAGGTGTCTC
TP53_856-F	CTTTGAGGTGCGTGTTTGTGC
TP53_856-R	CCTGGGGGCAGCTCGTGG
M13F(-47)	CGCCAGGGTTTTCCCAGTCACGAC
M13R(-48)	AGCGGATAACAATTTCACACAGGA
T7-AasgRNA-F	TAATACGACTCACTATAGGGTCTAAAGGACAGAATTTTTCAACGGGTG
T7-AasgRNA-M13mp18-R	TCATAGTTAGCGTAACGATCGTGCCACTTCGCTTGAAGTTCA
T7-AasgRNA-Target 1-R	ATAGCCAAGGGACTGGGAGGGTGCCACTTCGCTTGAAGTTCA
T7-AasgRNA-Target 2-R	ACTGAAGCCATTCCTCTCCAGTGCCACTTCGCTTGAAGTTCA
T7-AasgRNA-CaMV-R	AGGTTTCGCGGCGAAGATAGGTGCCACTTCGCTTGAAGTTCA
T7-AasgRNA-B101-R	TCCACGCCCACATGGTCGCGGTGCCACTTCGCTTGAAGTTCA
T7-AatgRNA-B101-R	TCCACGCCCATATGGTCGCGGTGCCACTTCGCTTGAAGTTCA
T7-AasgRNA-O01-R	GGTAGACCATCCTGCAAGCGGTGCCACTTCGCTTGAAGTTCA
T7-AatgRNA-O01-R	GGTAGACCATCCTGCAAGTGGTGCCACTTCGCTTGAAGTTCA
T7-AasgRNA-O02/03-R	CAGGATGGTCTACCCCCAGTGTGCCACTTCGCTTGAAGTTCA
T7-AatgRNA-O02/03-R	CAGGATGGTCTACCCCCATTGTGCCACTTCGCTTGAAGTTCA

T7-AasgRNA-BRCA1_3232-R	ATTGCTTGAGCTGGCTCCTTGTGCCACTTCGCTTGAAGTTCA
T7-AatgRNA-BRCA1_3232-1-R	ATTGCTTGAGCTGGCTCCTAGTGCCACTTCGCTTGAAGTTCA
T7-AatgRNA-BRCA1_3232-2-R	ATTGCTTGAGCTGGGTCCTTGTGCCACTTCGCTTGAAGTTCA
T7-AatgRNA-BRCA1_3232-3-R	ATTGCTTGACCTGGCTCCTTGTGCCACTTCGCTTGAAGTTCA
T7-AatgRNA-BRCA1_3232-4-R	ATTGGTTGAGCTGGCTCCTTGTGCCACTTCGCTTGAAGTTCA
T7-AasgRNA-BRCA1_3537-R	CCACTTCCCATAGGCTGTTCGTGCCACTTCGCTTGAAGTTCA
T7-AatgRNA-BRCA1_3537-1-R	CCACTTCCCATAGGCTGTTGGTGCCACTTCGCTTGAAGTTCA
T7-AatgRNA-BRCA1_3537-2-R	CCACTTCCCATAGGGTGTTCGTGCCACTTCGCTTGAAGTTCA
T7-AatgRNA-BRCA1_3537-3-R	CCACTTCCCTTAGGCTGTTCGTGCCACTTCGCTTGAAGTTCA
T7-AatgRNA-BRCA1_3537-4-R	CCACATCCCATAGGCTGTTCGTGCCACTTCGCTTGAAGTTCA
T7-AasgRNA-HPV16_site_1-R	TTTGTTAGCCTGTAATGTAGGTGCCACTTCGCTTGAAGTTCA
T7-AasgRNA-HPV18_site_1-R	TTTAGTATCTTGCAATGTACGTGCCACTTCGCTTGAAGTTCA
T7-AasgRNA-HPV16_site_2-R	GTGCTGCCATATCTACTTCAGTGCCACTTCGCTTGAAGTTCA
T7-AasgRNA-HPV18_site_2-R	TGTGTAGAAGCACATATTGTGTGCCACTTCGCTTGAAGTTCA
T7-AasgRNA-TP53_856-R	GACCGGCGCACAGAGAAAGAGTGCCACTTCGCTTGAAGTTC
T7-AatgRNA-TP53_856-1-R	GACCGGCGCACAGAGAAATAGTGCCACTTCGCTTGAAGTTC
T7-AatgRNA-TP53_856-2-R	GACCGGCGCACACAGAAAGAGTGCCACTTCGCTTGAAGTTC
T7-AatgRNA-TP53_856-3-R	GACCGGCGCTCAGAGAAAGAGTGCCACTTCGCTTGAAGTTC
T7-AasgRNA-target_1-R	GGGTGGTGCCCATCCTGGTCGTGCCACTTCGCTTGAAGTTCA
T7-AasgRNA-target_2-R	TCATAGTTAGCGTAACGATCGTGCCACTTCGCTTGAAGTTCA
T7-AasgRNA-target_3-R	TCAGCTGTGGAACACCCAGGGTGCCACTTCGCTTGAAGTTCA
T7-AasgRNA-target_4-R:	GCCTTATTAAATGACTTCTCGTGCCACTTCGCTTGAAGTTCA

T7-AasgRNA-DNMT1_target_1-R:	TTCACCGAGCAGGAGTGAGGGTGCCACTTCGCTTGAAGTTCA
T7-AasgRNA-DNMT1_target_2-R	CACGGAGACTGAACACTCCTGTGCCACTTCGCTTGAAGTTCA
T7-AasgRNA-DNMT1_target_3-R:	TAACAGACATGGACCATCAGGTGCCACTTCGCTTGAAGTTCA
T7-AasgRNA-DNMT1_target_4-R	TTATTTTAGCTGAAGGGAAAGTGCCACTTCGCTTGAAGTTCA
T7-AasgRNA-EMX1_target_1-R	GGAGGGAGGGGCACAGATGAGTGCCACTTCGCTTGAAGTTCA
T7-AasgRNA-EMX1_target_2-R	GTGGTTCCAGAACCGGAGGAGTGCCACTTCGCTTGAAGTTCA
T7-AasgRNA-HERC2A-R:	AGAACTTGACATTTAATGCTGTGCCACTTCGCTTGAAGTTCA
T7-AasgRNA-HERC2G-R:	AGAACTTGACACTTAATGCTGTGCCACTTCGCTTGAAGTTCA
BRCA1_3232-sgPOT-1-F	CTGCTCCCCTCATGGCCCCACTTGCTCCTT
BRCA1_3232-sgPOT-1-R	ACTATGTTTCTAAGATGACATAAAGGTGGG
BRCA1_3232-sgPOT-2-F	ATCATGGGAACTGCAGGAAGAGGTTCCCAG
BRCA1_3232-sgPOT-2-R	AATGTCAGATGCACAATTACTAGCTCTTTG
BRCA1_3232-sgPOT-3-F	GATTTTTATGGAGGTTGACTTTGTGTGTGC
BRCA1_3232-sgPOT-3-R	AGCTGCACTGAGAACCATGTTTCCCTCCTC
BRCA1_3232-sgPOT-4-F	GTCTGTCTATAGGGGTTGGGGGAGAAAGAA
BRCA1_3232-sgPOT-4-R	ATACCAGCCTATGGATTTCGGACTTGCAAG
BRCA1_3232-sgPOT-5-F	AAGATAACAATGCCCATGAGTGTGGCAGGG
BRCA1_3232-sgPOT-5-R	TGGTTGCCATTCTCCTCGGCCCCCACCCAG
BRCA1_3232-sgPOT-6-F	TATTCACTTCATGTAGGTGCTGAATTTAAT
BRCA1_3232-sgPOT-6-R	TGGCTCTGAATGGGAATCCACGGCTTCCTT

BRCA1_3232-sgPOT-7-F	AAGAATTTCAGCTGAATAACAACAAAAAAG
BRCA1_3232-sgPOT-7-R	TAGTATAAATACTTAAAGCTATAAATTTTT
BRCA1_3232-sgPOT-8-F	TTACAGATGCTTCCTCAGCAACGGAAAGCC
BRCA1_3232-sgPOT-8-R	AACACACTGGTCACCTGTGGAATTTCTGCT
BRCA1_3232-sgPOT-9-F	CTCACAAAGATTACAAACCACAAAAGGAAA
BRCA1_3232-sgPOT-9-R	GAGATTGCTATGTTCAGTTTTGGGGTATAC
BRCA1_3232-sgPOT-10-F	ATTTTGAACTGAATGAAAATGAAAACGCCA
BRCA1_3232-sgPOT-10-R	TTATAAAAAACATTTAATGCCAGAACATTT
BRCA1_3232-sgPOT-11-F	ACCCTGACTGATACAGATAACCTAGCCCAA
BRCA1_3232-sgPOT-11-R	ATAGTTTAATAGTGGAGCAACTGTTAGAAT
BRCA1_3232-sgPOT-12-F	GCACCATATGGAAGTTATCAAGGCTTATAG
BRCA1_3232-sgPOT-12-R	ATCCCAGCTGTTCAGCTGTGGTTCAAGGGG
BRCA1_3232-sgPOT-13-F	GCAGCCGGGTTCTGCCCTCGTTCGGGGAAC
BRCA1_3232-sgPOT-13-R	ACTGAAGGCTCAAGAGGCAGCCTCTGAGGA
BRCA1_3232-sgPOT-14-F	CATATCATTGCTGAGTATTCCTTTGGAGAT
BRCA1_3232-sgPOT-14-R	TCACTGGACATGAGCTGCTCTTGAGAATAA
BRCA1_3232-sgPOT-15-F	TTCTAGTTGTTTTCTCTTTTGGAAATACAT
BRCA1_3232-sgPOT-15-R	ATATACACTTCCTCATAAGGATTACAGTGA
BRCA1_3232-sgPOT-17-F	AATATGCTGGAGATTTATGGGGACTATCTA
BRCA1_3232-sgPOT-17-R	TTTATATGGCTTGACTGACTCTTTTACCAA
BRCA1_3232-sgPOT-18-F	ATGTCACAAACGTTGTGCAGTCCCTATGAG
BRCA1_3232-sgPOT-18-R	CCGAAGGGTATTTATCAAGCTTTGAAGTTT

BRCA1_3232-sgPOT-19-F	ACAATTGTTATAGGGAAGGCAAAATTCCAC
BRCA1_3232-sgPOT-19-R	TATGCTCTTATTCATTTTAATCTATCTTAT
BRCA1_3232-sgPOT-20-F	GACCTAGCTCCTAGGATGCTTGAGGCTGAA
BRCA1_3232-sgPOT-20-R	GTTTCAGCTACTCTAATTCTACCTGACTGG
BRCA1_3232-sgPOT-21-F	CTTAGGAAGCTCCAAGTTATAGAACCTTCT
BRCA1_3232-sgPOT-21-R	ATACCAAGATCTTGGCTCCTGTCTTAAGGA
BRCA1_3232-tgPOT-1-F	GAAAGGTAAATGCTAGGAACAGGTGGCATC
BRCA1_3232-tgPOT-1-R	ATAGTTTGTTTACCTACCTATAACATAAAG
BRCA1_3232-tgPOT-2-F	CTTTTAGATAAATGGAAATGAAGACAAACA
BRCA1_3232-tgPOT-2-R	TATAATAATAATAAAATTAAAAAAAAATTC
BRCA1_3232-tgPOT-7-F	TTTGGAGCAAGTACCAAATGCCAAGCACTG
BRCA1_3232-tgPOT-7-R	TCATCTGTAAAACACCACCTTTTTGAGTTT
BRCA1_3232-tgPOT-8-F	ATGATTGCCCGCCAAGATTTTCCTTTTTTT
BRCA1_3232-tgPOT-8-R	CCTGTAATCTCAGCTACTTGGGAGGCTGAG
BRCA1_3232-tgPOT-9-F	TGCGTTGGGCAAACACAAAGATGCTATTTG
BRCA1_3232-tgPOT-9-R	TTATCACAGGCACCTCACGAGTTAGGTGAG
BRCA1_3232-tgPOT-10-F	AAAGCCTGGGAGTGATTAACAACCACTGCA
BRCA1_3232-tgPOT-10-R	CTTACAGGAGCTTTTAAGTCCACAATTTCC
BRCA1_3232-tgPOT-11-F	GAAAATTTCTGAAATGAATTAAAATGAGAA
BRCA1_3232-tgPOT-11-R	TGAATACTAATATAATACTATGAATTTCTC
BRCA1_3232-tgPOT-12-F	GCTGCAGAGCCGGTGGTTTTCCTATCACTA
BRCA1_3232-tgPOT-12-R	TGCATCCGCGTCAGGTAAGGACGCCCGGTT

BRCA1_3232-tgPOT-13-F	CCTTTCAAAGAGTGCATTTGCTATCACAAA
BRCA1_3232-tgPOT-13-R	GATTCTTCATGTTTCATTTGGGGTAATAAG
BRCA1_3232-tgPOT-14-F	AATATTTTGAACTGAATGAAAATGAAAAAA
BRCA1_3232-tgPOT-14-R	CTAATATAAGCATTTAATGCTGTCGGTTTC
BRCA1_3232-tgPOT-15-F	AAATTATTTTAACCTGAATGATAATGAAAA
BRCA1_3232-tgPOT-15-R	TCTAAAATATACATATAAGGCTATATATTT
BRCA1_3232-tgPOT-16-F	TTTTGAATTAAATAAAAATGATACACAGCT
BRCA1_3232-tgPOT-16-R	TCTAAATAAGCATTTCAAACTATAAATTCC
BRCA1_3232-tgPOT-18-F	TTTTGTTTGAGTGATAATAAATATATTGTA
BRCA1_3232-tgPOT-18-R	GAATATATGCATTTAACACTATATATTTTC
BRCA1_3232-tgPOT-19-F	TCTCTCTCTTTCTTTCCAAGAGGCCAATTT
BRCA1_3232-tgPOT-19-R	GCTGATGAGGGGGTTAGAAAAAGCTTTGTG
BRCA1_3232-tgPOT-20-F	GGTTAGCTGGATTTGTCCGTATTGCAGATG
BRCA1_3232-tgPOT-20-R	GAAAAGGTGATGCATGGCACCTGTGCAGAG
BRCA1_3232-tgPOT-21-F	TAATGCATAACTTCATTTAAAAAGTTTTGA
BRCA1_3232-tgPOT-21-R	TCCATAGCATACAGACTATTTTAACAATCT
BRCA1_3232-tgPOT-22-F	CATTTTGTTCTGAATAAAAAGAAGACTACA
BRCA1_3232-tgPOT-22-R	TTTAACATGTGTTTAAAACTATAAATTCCC
BRCA1_3232-tgPOT-23-F	TCGAACATACCCAAGAGTAGAGAGAACCAT
BRCA1_3232-tgPOT-23-R	AGGGGAGGGATACATGAAACAGGATGGCAA
BRCA1_3232-tgPOT-24-F	TATATTGAACTGAAAGAAAAGGAAAATACA
BRCA1_3232-tgPOT-24-R	TGATCTAAGCATTTGGTGCTGTAAATTTCT

TP53_856-sgPOT-1-F	AAAGTTATTTGGAATATTTCTGCTCGACAG
TP53_856-sgPOT-1-R	TTTAGCTGGCATCACTGGCGTCCTGAAGAT
TP53_856-sgPOT-2-F	GAGGAGTTTGACTTTAGCATTAGCACATGA
TP53_856-sgPOT-2-R	ATAGAGTTTACTTGTGCTATATTCCTATTG
TP53_856-sgPOT-3-F	TGGCTCCCCTCTGTCCATCTGCAAGACAGA
TP53_856-sgPOT-3-R	AGGCACTCCTGGGCTCAGCTTGACATGGCT
TP53_856-sgPOT-4-F	GCACCTCCACGGCACTCCCAACTTTTTGTC
TP53_856-sgPOT-4-R	CGTCCCAACTCCTGCGCACGAAGGTGTCGC
TP53_856-sgPOT-5-F	TGGAACTTGCCAACAGATTTTTGCTTTTTG
TP53_856-sgPOT-5-R	GAAAATAGCAGCATGGGCAGCTGGCAGAGG
TP53_856-sgPOT-6-F	CAGCCAAAAACAGCAGTTCATTTGGGCATG
TP53_856-sgPOT-6-R	GCTGGTGTGAGCGTTGAACAGCTTGACCTG
TP53_856-sgPOT-7-F	GTTTATTTCTGGGTTTTATATTTGATTCTA
TP53_856-sgPOT-7-R	TAAGACTTATAATAAAGCAATTAAAATTAG
TP53_856-sgPOT-9-F	ATCTTAGGACTCACAGTCTCCGGGCCTTTC
TP53_856-sgPOT-9-R	AGAAAACAAGTAATCGTTCTGAGATTGAAA
TP53_856-sgPOT-10-F	GAACTCCACATGAGATCACAGGTGCAGGAT
TP53_856-sgPOT-10-R	TGGACCCATTGACAAGTGACTGAAACCCCG
TP53_856-sgPOT-11-F	GAACTCCACATGAGACCACAGGTGCAGGAT
TP53_856-sgPOT-11-R	TGGACCCATTGACAAGTGACTGAAACCCTG
TP53_856-tgPOT-2-F	CTGTAGAGAACTAATGTAGTCTACACTGTA
TP53_856-tgPOT-2-R	GTTACTTCACTGTGCAAGGCTCCTGAAAAT

TP53_856-tgPOT-3-F	CAACCCAACAGATTAGTTTGAAAACAATGT
TP53_856-tgPOT-3-R	GAGAATGTGCTAAGTCAGCTAGGCACCTGG
RPA-BRCA1_3232-F-1	GTACAGTGAGCACAATTAGCCGTAATAACA
RPA-BRCA1_3232-R-1	ACTTCATTAGTACTGGAACCTACTTCATTA
RPA-TP53_856-F	CTTTGAGGTGCGTGTTTGTGCCTGTCCTGG
RPA-TP53_856-R	CCTGGGGGCAGCTCGTGGTGAGGCTCCCCT
RPA-BRCA1_3232-F-2	ACATTCCAAGTACAGTGAGCACAATTAGCC
RPA-BRCA1_3232-R-2	ATTTTGGCCCTCTGTTTCTACCTAGTTCTG
**RNA**	
AasgRNA-M13mp18	GUCUAAAGGACAGAAUUUUUCAACGGGUGUGCCAAUGGCCACUUUCCAGGUGGCAAAGCCCGUUGAACUUCAAGCGAAGUGGCACGAUCGUUACGCUAACUAUGA
AasgRNA-Target 1	GUCUAAAGGACAGAAUUUUUCAACGGGUGUGCCAAUGGCCACUUUCCAGGUGGCAAAGCCCGUUGAACUUCAAGCGAAGUGGCACCCUCCCAGUCCCUUGGCUAU
AasgRNA-Target 2	GUCUAAAGGACAGAAUUUUUCAACGGGUGUGCCAAUGGCCACUUUCCAGGUGGCAAAGCCCGUUGAACUUCAAGCGAAGUGGCACUGGAGAGGAAUGGCUUCAGU
AasgRNA-CaMV	GUCUAAAGGACAGAAUUUUUCAACGGGUGUGCCAAUGGCCACUUUCCAGGUGGCAAAGCCCGUUGAACUUCAAGCGAAGUGGCACCUAUCUUCGCCGCGAAACCU
AasgRNA-HPV16_site_2	GUCUAAAGGACAGAAUUUUUCAACGGGUGUGCCAAUGGCCACUUUCCAGGUGGCAAAGCCCGUUGAACUUCAAGCGAAGUGGCACUGAAGUAGAUAUGGCAGCAC
AasgRNA-HPV18_site_2	GUCUAAAGGACAGAAUUUUUCAACGGGUGUGCCAAUGGCCACUUUCCAGGUGGCAAAGCCCGUUGAACUUCAAGCGAAGUGGCACACAAUAUGUGCUUCUACACA
AasgRNA-B101	GUCUAAAGGACAGAAUUUUUCAACGGGUGUGCCAAUGGCCACUUUCCAGGUGGCAAAGCCCGUUGAACUUCAAGCGAAGUGGCACCGCGACCAUGUGGGCGUGGA
AatgRNA-B101	GUCUAAAGGACAGAAUUUUUCAACGGGUGUGCCAAUGGCCACUUUCCAGGUGGCAAAGCCCGUUGAACUUCAAGCGAAGUGGCACCGCGACCAUAUGGGCGUGGA
AasgRNA-O01	GUCUAAAGGACAGAAUUUUUCAACGGGUGUGCCAAUGGCCACUUUCCAGGUGGCAAAGCCCGUUGAACUUCAAGCGAAGUGGCACCGCUUGCAGGAUGGUCUACC

AatgRNA-001	GUCUAAAGGACAGAAUUUUUCAACGGGUGUGCCAAUGGCCACUUUCCAGGUGGCAAAGCCCGUUGAACUUCAAGCGAAGUGGCACCACUUGCAGGAUGGUCUACC
AasgRNA-002/03	GUCUAAAGGACAGAAUUUUUCAACGGGUGUGCCAAUGGCCACUUUCCAGGUGGCAAAGCCCGUUGAACUUCAAGCGAAGUGGCACACUGGGGGUAGACCAUCCUG
AatgRNA-002/03	GUCUAAAGGACAGAAUUUUUCAACGGGUGUGCCAAUGGCCACUUUCCAGGUGGCAAAGCCCGUUGAACUUCAAGCGAAGUGGCACAAUGGGGGUAGACCAUCCUG
AasgRNA-BRCA1_3232	GUCUAAAGGACAGAAUUUUUCAACGGGUGUGCCAAUGGCCACUUUCCAGGUGGCAAAGCCCGUUGAACUUCAAGCGAAGUGGCACAAGGAGCCAGCUCAAGCAAU
AatgRNA-BRCA1_3232-1	GUCUAAAGGACAGAAUUUUUCAACGGGUGUGCCAAUGGCCACUUUCCAGGUGGCAAAGCCCGUUGAACUUCAAGCGAAGUGGCACUAGGAGCCAGCUCAAGCAAU
AatgRNA-BRCA1_3232-2	GUCUAAAGGACAGAAUUUUUCAACGGGUGUGCCAAUGGCCACUUUCCAGGUGGCAAAGCCCGUUGAACUUCAAGCGAAGUGGCACAAGGACCCAGCUCAAGCAAU
AatgRNA-BRCA1_3232-3	GUCUAAAGGACAGAAUUUUUCAACGGGUGUGCCAAUGGCCACUUUCCAGGUGGCAAAGCCCGUUGAACUUCAAGCGAAGUGGCACAAGGAGCCAGGUCAAGCAAU
AatgRNA-BRCA1_3232-4	GUCUAAAGGACAGAAUUUUUCAACGGGUGUGCCAAUGGCCACUUUCCAGGUGGCAAAGCCCGUUGAACUUCAAGCGAAGUGGCACAAGGAGCCAGCUCAACCAAU
AasgRNA-BRCA1_3537	GUCUAAAGGACAGAAUUUUUCAACGGGUGUGCCAAUGGCCACUUUCCAGGUGGCAAAGCCCGUUGAACUUCAAGCGAAGUGGCACGAACAGCCUAUGGGAAGUGG
AatgRNA-BRCA1_3537-1	GUCUAAAGGACAGAAUUUUUCAACGGGUGUGCCAAUGGCCACUUUCCAGGUGGCAAAGCCCGUUGAACUUCAAGCGAAGUGGCACCAACAGCCUAUGGGAAGUGG
AatgRNA-BRCA1_3537-2	GUCUAAAGGACAGAAUUUUUCAACGGGUGUGCCAAUGGCCACUUUCCAGGUGGCAAAGCCCGUUGAACUUCAAGCGAAGUGGCACGAACACCCUAUGGGAAGUGG
AatgRNA-BRCA1_3537-3	GUCUAAAGGACAGAAUUUUUCAACGGGUGUGCCAAUGGCCACUUUCCAGGUGGCAAAGCCCGUUGAACUUCAAGCGAAGUGGCACGAACAGCCUAAGGGAAGUGG
AatgRNA-BRCA1_3537-4	GUCUAAAGGACAGAAUUUUUCAACGGGUGUGCCAAUGGCCACUUUCCAGGUGGCAAAGCCCGUUGAACUUCAAGCGAAGUGGCACGAACAGCCUAUGGGAUGUGG
crRNA-M13mp18	AUUUCUACUAUUGUAGAUGAUCGUUACGCUAACUAUGAGGG
crRNA-HPV16_site_2	AUUUCUACUAAGUGUAGAUUGAAGUAGAUAUGGCAGCACAUA
crRNA-HPV18_site_2	AUUUCUACUAAGUGUAGAUACAAUAUGUGCUUCUACACAGUC

sgRNA-M13mp18	GAUCGUUACGCUAACUAUGAGUUUUAGAGCUAGAAAUAGCAAGUUAAAAUAAGGCUAGUCCGUUAUCAACUUGAAAAAGUGGCACCGAGUCGGUGCU
T7-AasgRNA-HPV16_site_1	GGGUCUAAAGGACAGAAUUUUUCAACGGGUGUGCCAAUGGCCACUUUCCAGGUGGCAAAGCCCGUUGAACUUCAAGCGAAGUGGCACCUACAUUACAGGCUAACAAA
T7-AasgRNA-HPV18_site_1	GGGUCUAAAGGACAGAAUUUUUCAACGGGUGUGCCAAUGGCCACUUUCCAGGUGGCAAAGCCCGUUGAACUUCAAGCGAAGUGGCACGUACAUUGCAAGAUACUAAA
T7-AasgRNA-dsActivator-1	GGGUCUAAAGGACAGAAUUUUUCAACGGGUGUGCCAAUGGCCACUUUCCAGGUGGCAAAGCCCGUUGAACUUCAAGCGAAGUGGCACGACCAGGAUGGGCACCACCC
T7-AasgRNA-dsActivator-2	GGGUCUAAAGGACAGAAUUUUUCAACGGGUGUGCCAAUGGCCACUUUCCAGGUGGCAAAGCCCGUUGAACUUCAAGCGAAGUGGCACGAUCGUUACGCUAACUAUGA
T7-AasgRNA-dsActivator-3	GGGUCUAAAGGACAGAAUUUUUCAACGGGUGUGCCAAUGGCCACUUUCCAGGUGGCAAAGCCCGUUGAACUUCAAGCGAAGUGGCACGAGAAGUCAUUUAAUAAGGC
T7-AasgRNA-DNMT1-1	GGGUCUAAAGGACAGAAUUUUUCAACGGGUGUGCCAAUGGCCACUUUCCAGGUGGCAAAGCCCGUUGAACUUCAAGCGAAGUGGCACCCUCACUCCUGCUCGGUGAA
T7-AasgRNA-DNMT1-2	GGGUCUAAAGGACAGAAUUUUUCAACGGGUGUGCCAAUGGCCACUUUCCAGGUGGCAAAGCCCGUUGAACUUCAAGCGAAGUGGCACAGGAGUGUUCAGUCUCCGUG
T7-AasgRNA-DNMT1-3	GGGUCUAAAGGACAGAAUUUUUCAACGGGUGUGCCAAUGGCCACUUUCCAGGUGGCAAAGCCCGUUGAACUUCAAGCGAAGUGGCACCUGAUGGUCCAUGUCUGUUA
T7-AasgRNA-DNMT1-4	GGGUCUAAAGGACAGAAUUUUUCAACGGGUGUGCCAAUGGCCACUUUCCAGGUGGCAAAGCCCGUUGAACUUCAAGCGAAGUGGCACUUUCCCUUCAGCUAAAAUAA
T7-AasgRNA-EMX1-2	GGGUCUAAAGGACAGAAUUUUUCAACGGGUGUGCCAAUGGCCACUUUCCAGGUGGCAAAGCCCGUUGAACUUCAAGCGAAGUGGCACUCCUCCGGUUCUGGAACCAC
T7-AasgRNA-HERC2A	GGGUCUAAAGGACAGAAUUUUUCAACGGGUGUGCCAAUGGCCACUUUCCAGGUGGCAAAGCCCGUUGAACUUCAAGCGAAGUGGCACAGCAUUAAAUGUCAAGUUCU
T7-AasgRNA-HERC2G	GGGUCUAAAGGACAGAAUUUUUCAACGGGUGUGCCAAUGGCCACUUUCCAGGUGGCAAAGCCCGUUGAACUUCAAGCGAAGUGGCACAGCAUUAAGUGUCAAGUUCU
**FQ ssDNA reporter**	
poly_A	5'-FAM-AAAAA-BHQ1-3'
poly_T	5'-FAM-TTTTT-BHQ1-3'
poly_G	5'-FAM-GGGGG-BHQ1-3'

poly_C	5′-FAM-CCCCC-BHQ1-3′

[a] Target 1 and Target 2 are re-named from mouse *Nrl* and *Prmt7* target sites used in our previous works for mammalian genome editing [1], respectively.

[b] HPV16 and HPV18 (site 1 and site 2) and dsActivator 1 and 2 are derived and re-named from previous work used for Cas12a-DETECTR detection [2]. HPV16 and HPV18 (site 1) are varied by 6 base pairs. HPV16 and HPV18 (site 2) are sequences within the hypervariable loop C of the L1-encoding gene.

[c] HERC2 SNP (>A and >G) and dsActivator 3 are derived and re-named from previous work used for Cas14-DETECTR detection [3].

[d] *DNMT1* and *EMX1* sites are derived from previous work used for genome editing [4].

Table S2. The components of buffers tested for Cas12b-mediated DNA detection in this study. Buffer 1, 3, 4, 5 were derived from previous reports, and Buffer 2, 6, 7, 8 were derived from commercial buffers. 1: Nuclease Assay Buffer [5]; 2: NEBuffer™ 3.1; 3: Cas12a Binding Buffer [2]; 4: Cas13 Nuclease Assay Buffer [6]; 5: Cas12a Cleavage Buffer [2]; 6: NEBuffer™ 2; 7: NEBuffer™ 2.1; 8: Cutsmart® Buffer.

Buffer ID	1x Components
1	60 mM NaCl, 40 mM Tris-HCl, 6 mM $MgCl_2$, pH7.5
2	100 mM NaCl, 50 mM Tris-HCl, 10 mM $MgCl_2$, 100 µg/ml BSA, pH 7.9
3	20 mM Tris-HCl, 5 mM $MgCl_2$, 100 mM KCl, 1 mM DTT, 5% Glycerol, 50 µg/mL Heparin
4	60 mM NaCl, 20 mM HEPES, 6 mM $MgCl_2$, pH6.8
5	150 mM KCl, 20 mM HEPES, 10 mM $MgCl_2$, 0.5 mM DTT, 1% Glycerol, pH7.5
6	50 mM NaCl, 10 mM Tris-HCl, 10 mM $MgCl_2$, 1 mM DTT, pH7.9
7	50 mM NaCl, 10 mM Tris-HCl, 10 mM $MgCl_2$, 100 µg/ml BSA, pH 7.9
8	50mM Potassium Acetate, 20mM Tris-acetate, 10mM Magnesium Acetate, 100µg/ml BSA, pH 7.9
9	50 mM Tris-HCl, 10 mM $MgCl_2$, 10 mM DTT, pH 7.5
10	66 M Tris-HCl, 10 mM $MgCl_2$, 1 mM DTT ,7.5% PEG6000, pH7.6

References

1. Teng F, Cui T, Feng G, Guo L, Xu K, Gao Q, Li T, Li J, Zhou Q, Li W: **Repurposing CRISPR-Cas12b for mammalian genome engineering.** *Cell Discov* 2018, **4:**63.
2. Chen JS, Ma E, Harrington LB, Da Costa M, Tian X, Palefsky JM, Doudna JA: **CRISPR-Cas12a target binding unleashes indiscriminate single-stranded DNase activity.** *Science* 2018, **360:**436-439.
3. Harrington LB, Burstein D, Chen JS, Paez-Espino D, Ma E, Witte IP, Cofsky JC, Kyrpides NC, Banfield JF, Doudna JA: **Programmed DNA destruction by miniature CRISPR-Cas14 enzymes.** *Science* 2018, **362:**839-842.
4. Zetsche B, Gootenberg JS, Abudayyeh OO, Slaymaker IM, Makarova KS, Essletzbichler P, Volz SE, Joung J, van der Oost J, Regev A, et al: **Cpf1 is a single RNA-guided endonuclease of a class 2 CRISPR-Cas system.** *Cell* 2015, **163:**759-771.
5. Gootenberg JS, Abudayyeh OO, Lee JW, Essletzbichler P, Dy AJ, Joung J, Verdine V, Donghia N, Daringer NM, Freije CA, et al: **Nucleic acid detection with CRISPR-Cas13a/C2c2.** *Science* 2017, **356:**438-442.
6. Gootenberg JS, Abudayyeh OO, Kellner MJ, Joung J, Collins JJ, Zhang F: **Multiplexed and portable nucleic acid detection platform with Cas13, Cas12a, and Csm6.** *Science* 2018, **360:**439-444.
