# Supplementary material for: CDetection: CRISPR-Cas12b-based DNA detection with sub-attomolar sensitivity and single-base specificity
Source: Genome Biol. 2019 Jul 1;20:132. doi: 10.1186/s13059-019-1742-z (PMC6604390; doi:10.1186/s13059-019-1742-z)
Supplement: Supplementary file 3 — Review history. (DOCX 1323 kb) [file 13059_2019_1742_MOESM3_ESM.docx]

Review History

**Comments to author and authors’ point-by-point responses to the reviewers’ comments:**

**Reviewer 1**

In this manuscript the authors have developed a Cas12b-based DNA detection platform (CDetection) which shows higher sensitivity compared to the previously reported Cas12a. The authors also optimized tuned guide RNA (tgRNA) and showed that some tgRNAs can distinguish single base differences. Overall this manuscript is solid and the data support the conclusions. However, CRISPR has been previously used for DNA detection (cited by the authors). The novelty of the presented approach is the use of AaCas12b recently published by the authors. Several questions need to be addressed in the major revision.

*We thank the reviewers for their insightful comments and suggestions, which have helped us to improve the manuscript significantly. Below are the point-by-point responses following all the reviewers’ comments. We sincerely appreciate the positive comments and constructive suggestions of this reviewer, and our point-by-point responses are as follows.*

1. A major caveat is that tgRNA may have off-target sites in the genome. The authors showed that some sgRNA or tgRNAs can tolerate single base mismatches. Can off-target sites cause false positive signal? This critical issue must be addressed. What's the off-target profiles of some tgRNAs used in this study? Using experimental methods (eg, Guide-Seq) to find bona fide off-target sites and compare off-target sites in the DNA detection assay.

*We thank the reviewer’s helpful suggestions. As we mentioned in our mismatch evaluation, single or two continuous base mismatches can be tolerated to some degree (Figures 1a,b). To evaluate the off-target effect, we used Cas-OFFinder (Bae et al., 2014), an off-target prediction tool, to predict all potential off-target sites containing 1 to 3 mismatches with the sgRNA or tgRNA target sites, and synthesized the double-stranded DNAs covering all the off-target sequences as substrates for CDetection (see Table S1 in the revised manuscript). The results showed that DNA substrates from both sgRNA- and tgRNA-predicted off-targets could cause significant detection signals, suggesting that both sgRNA and tgRNA exhibited mismatch tolerance pattern (see the figure below, and Additional file 1: Figure S9a, b in the revised manuscript) as observed in our experiments (Fig. 1a, b) and in previous studies (Chen et al., 2018). This can be explained by the intrinsic features of all reported CRISPR-Cas systems that exhibit mismatch tolerance during target recognition (Cong et al., 2013; Fu et al., 2013; Gootenberg et al., 2017; Harrington et al., 2018; Hsu et al., 2013; Jinek et al., 2012; Kim et al., 2016; Kleinstiver et al., 2016; Strecker et al., 2019; Zetsche et al., 2015). To eliminate the off-target-associated undesired false positive signals, we specifically amplified the on-target sequence by RPA before CDetection, and the result showed that the false signals caused by potential off-targets were significantly reduced to a minimal level in tgRNA-based CDetection (see the figure below, and Additional file 1: Figure S9c, d in the revised manuscript). Collectively, our resulted showed that potential off-target DNAs bearing mismatches with on-target DNAs could cause false positive signals, however, these signals can be largely eliminated in real practice of CDetection through optimizing the tgRNA sequences or performing RPA with specific primers that can only amplify the on-target sites. Consistent with previous reports, DNA detection by all CRISPR-based detection tools need to rationally design and test the tgRNA and RPA primers for each substrate before put into use to ensure the minimal false positive signal (Gootenberg et al., 2017; Myhrvold et al., 2018). We have also added these comments in the Discussion part of the revised manuscript.*

***Figure S9 Potential off-target analysis of CDetection. a-b*** *Maximum fluorescence signal obtained from sgRNA- and tgRNA-based CDetection using potential off-targets without RPA pre-amplification. AaCas12b is incubated with sgRNA or tgRNA targeting their cognate synthetic* ***a*** *BRCA1 3232A>G or* ***b*** *TP53 856 G>A off-target dsDNAs individually. Potential off-target sites bearing 1 to 3 mismatches are predicted using Cas-OFFinder. POT, potential off-target; POTcom, common POTs predicted from both sgRNA and tgRNA. Error bars indicate standard errors of the mean (s.e.m.), n = 3.* ***c-d*** *Maximum fluorescence signal obtained from sgRNA or tgRNA-based CDetection using mixed off-targets after RPA pre-amplification. All potential off-targets from sgRNA and tgRNA targeting* ***c*** *BRCA1 3232A<G or* ***d*** *TP53 G>A are mixed together and amplified using RPA. POTmix, mixture of all potential off-targets of sgRNA and tgRNA. POTmix+wt, mixture of POTmix and wild-type on-target DNA substrate. POTmix+mut, mixture of POTmix and mutant on-target DNA substrate. Error bars indicate (s.e.m.), n = 3. Two-tailed Student’s t test is used for significance analysis. **P < 0.01; ***P < 0.001; ****P < 0.0001; ns, no significance.*

2. The authors showed that some cancer mutation-specific tgRNA can still be partially activated by WT DNA (BRCA1 3237A>G in Fig. S7d). Many oncogene mutations are heterozygous. What's the detection threshold of BRAC1 3237A>G mixed 1:1 with wildtype BRAC1 dsDNA?

*We appreciate the reviewer’s suggestion. We conducted CDetection of BRCA1_3232A>G single-base mutation in the human cells carrying this mutation using tgRNA-1. Our result suggests CDetection is able to discriminate single-nucleotide mutation between homozygous or heterozygous mutant DNAs and wildtype DNAs (see figure below, Figure 1 to reviewer). So we indicate that the CDetection strategy is able to discriminate SNP between homozygous or heterozygous mutations and wild-type sequences, which is also achievable for the SHERLOCK (Gootenberg et al., 2017). And we hope to focus on this point in our further works by optimizing this platform with rational design to enable quantitative analysis for molecular diagnostics.*

*Revised Fig. S8*

*e Maximum fluorescence signal showing the sensitivity for human BRCA1_3232A>G detection using selected tgRNA (BRCA1_3232A>G tgRNA-1). Mutant DNAs (BRCA1_3232A>G), wildtype DNAs (BRCA1_3232WT) and mixtures (mutant:WT = 1:1, 1:10 and 1:100) are used in CDetection analysis. BRCA1 wild-type genomic DNAs or 3232A>G mutant genomic DNAs are extracted from cell lines. Error bars indicate standard errors of the mean (s.e.m.), n = 3. Two-tailed Student’s t test is used for significance analysis among each other. **P < 0.01; ***P < 0.001; ****P < 0.0001.*

3. Fig. 2b-c cancer mutation detection. The authors used dsDNA diluted in human plasma as activator. A more stringent activator should be cellular genomic DNA. The circulation DNA from cancer patients likely contain genomic sequences. Although human plasma was used to dilute the dsDNA in this study, it's unclear whether the plasma donor can represent cancer patient plasma with circulating tumor genomic DNA. Using genomic DNA from BRAC1 mutant cancer cell line diluted in background genomic DNA is required to add translational value of this study.

*We thank the reviewer for the practical comment and suggestion. To explore the application of CDetection for cellular genomic DNAs, we used previously selected tgRNA (BRCA1_3232A>G tgRNA-1) and RPA to detect single base mutation in two breast tumor cell lines, SUM1315MO2 (carrying a BRCA1_3232A>G mutation) and MDA-MB-468 (BRCA1_3232WT). The cells are crudely extracted by direct digestion using Mouse Direct PCR Kit (Bimake). The result shows that tgRNA-1 can efficiently discriminate the SNP between different genotypes of cells (see the figure below, Fig. 2d and Additional file 1: Figure S8d in the revised manuscript).*

*Revised Figure 2*

*d Fluorescence time courses showing the sensitivity and specificity of CDetection with RPA for human BRCA1 3232A>G mutation detection using tgRNA (3232-1). BRCA1 wild-type genomic DNAs or 3232A>G mutant genomic DNAs are extracted from cell lines. Error bars indicate (s.e.m.), n = 3.*

*Revised Figure S8*

*d Maximum fluorescence signal obtained from time courses in Fig. 2d. CDetection is conducted using RPA-amplified dsDNAs from genomic DNAs or genomic DNAs without RPA-amplification directly. BRCA1 wild-type genomic DNAs or 3232A>G mutant genomic DNAs are extracted from cell lines. Error bars indicate (s.e.m.), n = 3. Two-tailed Student’s t test is used for significance analysis among each other in group. ***P < 0.001; ****P < 0.0001; ns, no significance.*

Minor points:

1. Methods "Statistical analysis was performed in SigmaPlot (version 1.0)." p value was not provided.

*We thank the reviewer’s meticulous comment. We have reformed the figures in the revised manuscript with statistical analysis.*

2. Please check the reference list: "Ref 6. (!!! INVALID CITATION !!! 4)." Ref 7 and 20 are the same paper from the authors' lab.

*We thank the reviewer’s delicate comment. We have corrected the reference list in the revised manuscript.*

*References*

*Bae, S., Park, J., and Kim, J.S. (2014). Cas-OFFinder: a fast and versatile algorithm that searches for potential off-target sites of Cas9 RNA-guided endonucleases. Bioinformatics 30, 1473-1475.*

*Chen, J.S., Ma, E., Harrington, L.B., Da Costa, M., Tian, X., Palefsky, J.M., and Doudna, J.A. (2018). CRISPR-Cas12a target binding unleashes indiscriminate single-stranded DNase activity. Science 360, 436-439.*

*Cong, L., Ran, F.A., Cox, D., Lin, S., Barretto, R., Habib, N., Hsu, P.D., Wu, X., Jiang, W., Marraffini, L.A., et al. (2013). Multiplex genome engineering using CRISPR/Cas systems. Science 339, 819-823.*

*Fu, Y., Foden, J.A., Khayter, C., Maeder, M.L., Reyon, D., Joung, J.K., and Sander, J.D. (2013). High-frequency off-target mutagenesis induced by CRISPR-Cas nucleases in human cells. Nat Biotechnol 31, 822-826.*

*Gootenberg, J.S., Abudayyeh, O.O., Lee, J.W., Essletzbichler, P., Dy, A.J., Joung, J., Verdine, V., Donghia, N., Daringer, N.M., Freije, C.A., et al. (2017). Nucleic acid detection with CRISPR-Cas13a/C2c2. Science 356, 438-442.*

*Harrington, L.B., Burstein, D., Chen, J.S., Paez-Espino, D., Ma, E., Witte, I.P., Cofsky, J.C., Kyrpides, N.C., Banfield, J.F., and Doudna, J.A. (2018). Programmed DNA destruction by miniature CRISPR-Cas14 enzymes. Science 362, 839-842.*

*Hsu, P.D., Scott, D.A., Weinstein, J.A., Ran, F.A., Konermann, S., Agarwala, V., Li, Y., Fine, E.J., Wu, X., Shalem, O., et al. (2013). DNA targeting specificity of RNA-guided Cas9 nucleases. Nat Biotechnol 31, 827-832.*

*Jinek, M., Chylinski, K., Fonfara, I., Hauer, M., Doudna, J.A., and Charpentier, E. (2012). A programmable dual-RNA-guided DNA endonuclease in adaptive bacterial immunity. Science 337, 816-821.*

*Kim, D., Kim, J., Hur, J.K., Been, K.W., Yoon, S.H., and Kim, J.S. (2016). Genome-wide analysis reveals specificities of Cpf1 endonucleases in human cells. Nat Biotechnol 34, 863-868.*

*Kleinstiver, B.P., Tsai, S.Q., Prew, M.S., Nguyen, N.T., Welch, M.M., Lopez, J.M., McCaw, Z.R., Aryee, M.J., and Joung, J.K. (2016). Genome-wide specificities of CRISPR-Cas Cpf1 nucleases in human cells. Nat Biotechnol 34, 869-874.*

*Myhrvold, C., Freije, C.A., Gootenberg, J.S., Abudayyeh, O.O., Metsky, H.C., Durbin, A.F., Kellner, M.J., Tan, A.L., Paul, L.M., Parham, L.A., et al. (2018). Field-deployable viral diagnostics using CRISPR-Cas13. Science 360, 444-448.*

*Strecker, J., Jones, S., Koopal, B., Schmid-Burgk, J., Zetsche, B., Gao, L., Makarova, K.S., Koonin, E.V., and Zhang, F. (2019). Engineering of CRISPR-Cas12b for human genome editing. Nat Commun 10, 212.*

*Zetsche, B., Gootenberg, J.S., Abudayyeh, O.O., Slaymaker, I.M., Makarova, K.S., Essletzbichler, P., Volz, S.E., Joung, J., van der Oost, J., Regev, A., et al. (2015). Cpf1 is a single RNA-guided endonuclease of a class 2 CRISPR-Cas system. Cell 163, 759-771.*

**Reviewer 2**

This is an interesting manuscript in which the authors developed a method for sensitive detection of nucleic acids using Cas12b. Overall, I believe the findings are important and the technology developed has potential for becoming a point-of-care diagnostic test. I have a few comments for the authors.

*We thank the reviewer for appreciating the value of our work, and our point-by-point responses are as follows.*

Major points:

1. As the authors noted, several methods have been developed for detecting nucleic acids. I am not convinced by the data presented that Cas13b is more sensitive than other methods. Gootenberg et al. also demonstrated sensitivity in the aM range and drawing conclusions based on analysis of a few, presumably carefully selected, targets is not enough. The authors should perform these comparisons at multiple targets randomly selected and clearly outline in which cases Cas12b provides an advantage over other methods.

*We sincerely thank the reviewer for the insightful and constructive suggestions. Since Cas13 uniquely trans-cleave ssRNA target, the Cas13-SHERLOCK enables dsDNA detection requiring an extra T7 in vitro transcription process. Our Cas12b-based CDetection is more similar to previously reported Cas12a-DETECTR method (Chen et al., 2018a) that does not require an extra transcription step for DNA detection, so we chose LbCas12a-DETECTR as a control and performed unbiased comparison between these two platforms. We chose 12 previously reported sites, including both detection sites and target sites (Chen et al., 2018a; Harrington et al., 2018; Zetsche et al., 2015) to analyze the sensitivity of these two platforms. Our results showed that AaCas12b behaves better than LbCas12a at 8 of the 12 sites; and except one site (DNMT1 site 2)* *that LbCas12a performed better, the rest 3 sites (EMX1 site 2, DNMT1 site 3 and BRCA1_3232G) showed no detectable signal by either method (see the figure below, Additional file 1: Figure S5 in the revised manuscript).*

*Figure S5 Comparison of sensitivities of AaCas12b- and LbCas12a-based DNA detection. (Left) Fluorescence time courses obtained from AaCas12b- and LbCas12a-based DNA detection with RPA pre-amplification. Cas12 proteins are incubated with their cognate gRNAs targeting synthetic dsDNAs with a final concentration of 10-16 M and 10-18 M. Error bars indicate standard errors of the mean (s.e.m.), n = 3. (Right) Maximum fluorescence signal obtained from timecourses in the left panel. Error bars indicate s.e.m., n = 3. Two-tailed Student’s t test is used for significance analysis between the two samples in group. *P < 0.05; **P < 0.01; ***P < 0.001; ****P < 0.0001; ns, no significance.*

2. The claim that sensitivity is in the aM range may not hold at other targets. Am I correct in interpreting that in Figure S4e the authors show that the sensitivity at target 2 is in the pM range? What is the mean sensitivity across targets and range of sensitivity that can be expected across multiple targets?

*We thank the reviewer for the helpful suggestion. Such is the case that the sensitivity at target 2 is in the pM range. Based on the results above (see the figure above, Additional file 1: Figure S5 in the revised manuscript), two-thirds of the tested targets achieve an aM sensitivity with one-third of the tested targets produce poor or even undetectable signals. Hence, these results combined with prior results in this work demonstrate CDetection is able to achieve attomolar sensitivity as the previous studies indicated (Chen et al., 2018a; Gootenberg et al., 2017).*

3. The authors claim that this detection system can be applied for detecting SNPs. However, the controlled conditions in which the experiments were performed do not resemble the experimental settings in which the experiments would be performed. The authors should demonstrate that their system can detect SNPs and mutations using cells or circulating DNA as start point rather than diluting template DNA into plasma.

*We thank the reviewer for the practical comment and suggestion. To explore the application of CDetection for cellular genomic DNAs, we used previously selected tgRNA (BRCA1_3232A>G tgRNA-1) and RPA to detect single base mutation in two breast tumor cell lines, SUM1315MO2 (carrying a BRCA1_3232A>G mutation) and MDA-MB-468 (BRCA1_3232WT). The cells are crudely extracted by direct digestion using Mouse Direct PCR Kit (Bimake). The result shows that tgRNA-1 can efficiently discriminate the SNP between different genotypes of cells (see the figure below, Fig. 2d and Additional file 1: Figure S8d in the revised manuscript).*

*Revised Figure 2*

*d Fluorescence time courses showing the sensitivity and specificity of CDetection with RPA for human BRCA1 3232A>G mutation detection using tgRNA (3232-1). BRCA1 wild-type genomic DNAs or 3232A>G mutant genomic DNAs are extracted from cell lines. Error bars indicate (s.e.m.), n = 3.*

*Revised Figure S8*

*d Maximum fluorescence signal obtained from time courses in Fig. 2d. CDetection is conducted using RPA-amplified dsDNAs from genomic DNAs or genomic DNAs without RPA-amplification directly. BRCA1 wild-type genomic DNAs or 3232A>G mutant genomic DNAs are extracted from cell lines. Error bars indicate (s.e.m.), n = 3. Two-tailed Student’s t test is used for significance analysis among each other in group. ***P < 0.001; ****P < 0.0001; ns, no significance.*

4. Since the authors propose that their system can be used for genotyping SNPs, what is the sensitivity for calling homozygous or heterozygous mutations?

*We appreciate the reviewer’s suggestion. We conducted CDetection of BRCA1_3232A>G single-base mutation in the human cells carrying this mutation using tgRNA-1. Our result* *suggests CDetection is able to discriminate single-nucleotide mutation between homozygous or heterozygous mutant DNAs and wildtype DNAs (see figure below, Figure 1 to reviewer). So we indicate that the CDetection strategy is able to discriminate SNP between homozygous or heterozygous mutations and wild-type sequences, which is also achievable for the SKERLOCK (Gootenberg et al., 2017). And we hope to focus on this point in our further works by optimizing this platform with rational design to enable quantitative analysis for molecular diagnostics.*

*Revised Fig. S8*

*e Maximum fluorescence signal showing the sensitivity for human BRCA1_3232A>G detection using selected tgRNA (BRCA1_3232A>G tgRNA-1). Mutant DNAs (BRCA1_3232A>G), wildtype DNAs (BRCA1_3232WT) and mixtures (mutant:WT = 1:1, 1:10 and 1:100) are used in CDetection analysis. BRCA1 wild-type genomic DNAs or 3232A>G mutant genomic DNAs are extracted from cell lines. Error bars indicate standard errors of the mean (s.e.m.), n = 3. Two-tailed Student’s t test is used for significance analysis among each other. **P < 0.01; ***P < 0.001; ****P < 0.0001.*

5. What is the background signal level in figure 1e?

*We thank the reviewer’s helpful suggestion. We are sorry for the mistake that we left out the negative control group using no activator. We have replaced the prior figure 1e with the new one (also see the figure below, Fig. 1e in the revised manuscript).*

*Revised Figure 1*

*e Maximum fluorescence signal obtained from AaCas12b-, PrCas12a- and LbCas12a-based DNA detection with RPA pre-amplification. Cas12 proteins are incubated with their cognate gRNA targeting a synthetic HPV16 dsDNA (site 2) mixed with background genome. Error bars indicate s.e.m., n = 3.*

6. The authors should discuss further the manuscript in Biorxiv and how it compares with the current technology described here.

*We thank the reviewer’s insightful comment. We have added further introduction and discussion among CDetection and other reported platforms in our revised manuscript.*

*There are mainly three differences between CDetection and HOLMESv2 (Li et al., 2018).*

*The two platforms base on two different Cas proteins. CDetection base on Alicyclobacillus acidiphilus NBRC 100859 (AaCas12b). While HOLMESv2 base on Alicyclobacillus acidoterrestris (AacCas12b). Our two platforms provide two more choices for nucleic acid detection field.*

*The two platforms base on two different isothermal amplification method. CDetection base on recombinase polymerase amplification (RPA), while HOLMESv2 base on Loop-Mediated Isothermal Amplification (LAMP). LAMP requires more pre-designed primers (6 primers), while RPA only need on pair of primer. What’s more, LAMP requires a longer incubation time (30 mins at 65* *℃), while RPA only need to incubate 5-10 mins at 37℃.*

*CDetection can identify single base mutation. While figures in HOLMESv2 didn’t* *show a solution to identify single base mutation.*

7. Statistical analysis for comparing samples is not provided.

*We thank the reviewer’s meticulous comment. We have replaced the figures in the revised manuscript with statistical analysis.*

Minor points:

1. A native speaker should review grammar and spelling as several sentences are difficult to understand.

*We thank the reviewer’s beneficial suggestion. We invited a gifted speaker helped us review grammar and spelling. We hope this time we made it more clear to understand.*

2. The authors profiled several fluorophore quencher labeled homopolymers. The motivation for these experiments should be described.

*We thank the reviewer’s suggestion. Sorry we didn’t make it clear in our original manuscript. Previous study have indicated that different Cas13 proteins show a diversity of dinucleotide cleavage motif preference (Gootenberg et al., 2018). So it’s important for us to evaluate the sequence preference of AaCas12b collateral trans ssDNA cleavage. It helps us to optimize the sensitivity of CDetection. Hence, we synthesized different fluorophore quencher labeled homopolymers, and found out that polyG-FQ could not be cleaved, polyC-FQ were difficult to cut, while polyA-FQ and polyT-FQ presented higher reporter signals. And we have added the reason for this motivation in our revised manuscript.*

3. Should figure 1b say target 2?

*Sorry we didn’t make it clear. Figure 1b evaluates how two continuous base mismatches influence the specificity of CDetection on target 1. In conclusion, we evaluated one or two continuous base mismatches influence, and found out a tolerance pattern.*

4. It would be helpful to provide diagrams of the target sites in the main figures describing the target sequences, PAMs, mismatches, etc.

*We thank the review’s kind suggestion. We have added the schematic diagram for describing the target sequences, PAMs, mismatches, etc. in our revised manuscript.*

5. Fig. 2b and 2c, the color code in the sequences should be described in the figure legend.

*We thank the review’s suggestion. We have added the corresponding description in Figure Legend in our revised manuscript.*

6. In figure S3b, the authors tested several buffers but the composition of those buffers was not indicated.

*Thanks for the review’s suggestion. We indeed have had the description for the composition of all buffers used in our work. We have listed the buffer components in Table S2 (see the table below, Additional file 2: Table S2 in the revised manuscript).*

*Revised Table S2*

*The components of buffers tested for Cas12b-mediated DNA detection in this study.*

| *Buffer ID* | *1x Components* |
| --- | --- |
| *1* | *60 mM NaCl, 40 mM Tris-HCl, 6 mM MgCl2, pH7.5* |
| *2* | *100 mM NaCl, 50 mM Tris-HCl, 10 mM MgCl2, 100 μg/ml BSA, pH 7.9* |
| *3* | *20 mM Tris-HCl, 5 mM MgCl2, 100 mM KCl, 1 mM DTT, 5% Glycerol, 50 µg/mL Heparin* |
| *4* | *60 mM NaCl, 20 mM HEPES, 6 mM MgCl2, pH6.8* |
| *5* | *150 mM KCl, 20 mM HEPES, 10 mM MgCl2, 0.5 mM DTT, 1% Glycerol, pH6.8* |
| *6* | *50 mM NaCl, 10 mM Tris-HCl, 10 mM MgCl2, 1 mM DTT, pH7.9* |
| *7* | *50 mM NaCl, 10 mM Tris-HCl, 10 mM MgCl2, 100 μg/ml BSA, pH 7.9* |
| *8* | *50mM Potassium Acetate, 20mM Tris-acetate, 10mM Magnesium Acetate, 100μg/ml BSA, pH 7.9* |
| *9* | *50 mM Tris-HCl, 10 mM MgCl2, 10 mM DTT, pH 7.5* |
| *10* | *M Tris-HCl, 10 mM MgCl2, 1 mM DTT ,7.5% PEG6000, pH7.6* |

*Buffer 1, 3, 4, 5 were derived from previous reports, and Buffer 2, 6, 7, 8 were derived from commercial buffers. 1: Nuclease Assay Buffer (Gootenberg et al., 2017); 2: NEBuffer™ 3.1; 3. Cas12a Binding Buffer (Chen et al., 2018b); 4: Cas13 Nuclease Aassay Buffer (Gootenberg et al., 2018); 5: Cas12a Cleavage Buffer (Chen et al., 2018b); 6: NEBuffer™ 2; 7: NEBuffer™ 2.1; 8: Cutsmart® Buffer.*

*References*

*Chen, J.S., Ma, E., Harrington, L.B., Da Costa, M., Tian, X., Palefsky, J.M., and Doudna, J.A. (2018a). CRISPR-Cas12a target binding unleashes indiscriminate single-stranded DNase activity. Science 360, 436-439.*

*Chen, J.S., Ma, E., Harrington, L.B., Da Costa, M., Tian, X., Palefsky, J.M., and Doudna, J.A. (2018b). CRISPR-Cas12a target binding unleashes indiscriminate single-stranded DNase activity. Science 360, 436.*

*Gootenberg, J.S., Abudayyeh, O.O., Kellner, M.J., Joung, J., Collins, J.J., and Zhang, F. (2018). Multiplexed and portable nucleic acid detection platform with Cas13, Cas12a, and Csm6. Science 360, 439-444.*

*Gootenberg, J.S., Abudayyeh, O.O., Lee, J.W., Essletzbichler, P., Dy, A.J., Joung, J., Verdine, V., Donghia, N., Daringer, N.M., Freije, C.A., et al. (2017). Nucleic acid detection with CRISPR-Cas13a/C2c2. Science 356, 438-442.*

*Harrington, L.B., Burstein, D., Chen, J.S., Paez-Espino, D., Ma, E., Witte, I.P., Cofsky, J.C., Kyrpides, N.C., Banfield, J.F., and Doudna, J.A. (2018). Programmed DNA destruction by miniature CRISPR-Cas14 enzymes. Science 362, 839-842.*

*Li, L., Li, S., and Wang, J. (2018). CRISPR-Cas12b-assisted nucleic acid detection platform. bioRxiv, 362889.*

*Zetsche, B., Gootenberg, J.S., Abudayyeh, O.O., Slaymaker, I.M., Makarova, K.S., Essletzbichler, P., Volz, S.E., Joung, J., van der Oost, J., Regev, A., et al. (2015). Cpf1 is a single RNA-guided endonuclease of a class 2 CRISPR-Cas system. Cell 163, 759-771.*
